# Supplementary material for: Continuous production of recombinant adeno-associated virus in the insect cell/baculovirus expression vector system
Source: Mol Ther Adv. 2026 Apr 29;34(2):201744. doi: 10.1016/j.omta.2026.201744 (PMC13195323; doi:10.1016/j.omta.2026.201744)
Supplement: Document S2. Article plus supplemental information [file mmc2.pdf]

Molecular Therapy
Advances
Original Article

Continuous production of recombinant adeno-associated virus in the insect cell/baculovirus expression vector system

John Joseph,[1,4] Francesco Destro,[2,4] Arella Yuan,[1,3] Daniel Antov,[1,3] Wenyu Chen,[1,3] Sally Song,[1,3] Matthew Theriault,[1,3] Chengcheng Yuan,[1,3] Alexander Sansom,[1,3] Chiara Lundin,[1,3] Tyler Burns,[1,3] Hadeel Abubaker Hamed,[1,3] Piero Morales,[1,3] Jessica Xinyuan Fan,[1,3] Avner Romero Rodriguez,[1,3] Sanyambe Sichuma,[1,3] Courtney McCracken,[1,3] Caleb Neufeld,[1] Jacqueline M. Wolfrum,[1] Prasanna Srinivasan,[1] Paul W. Barone,[1] Anthony J. Sinskey,[1,3] Richard D. Braatz,[1,2] and Stacy L. Springs[1]

[1]Center for Biomedical Innovation, Massachusetts Institute of Technology, Cambridge, MA, USA; [2]Department of Chemical Engineering, Massachusetts Institute of Technology, Cambridge, MA, USA; [3]Department of Biology, Massachusetts Institute of Technology, Cambridge, MA, USA

Continuous production processes may offer significant advantages for biotherapeutic manufacturing, including increased productivity, more consistent product quality, reduced facility footprint, and decreased process turnaround time. Despite these benefits, the *in situ* formation of defective recombinant baculovirus expression vectors (BEVs) hinders the continuous manufacturing of recombinant adeno-associated viruses (rAAV) in the baculovirus expression vector system. This study investigates an approach for reducing defective viruses through the infusion of standard recombinant baculoviruses (rBV) and the compartmentalization of early-stage and late-stage infected cells, resulting in stable rAAV production. In this study, rAAVs were continuously produced in a series of cascading reactors, comprising a feeder reactor, an infection reactor, and a production reactor. Residence times and transfer rates across the three reactors were optimized based on the production kinetics of rBV and rAAV derived from our mechanistic model. The majority of rBV was produced within the production reactor, thereby reducing the impact of defective viruses in the infection reactor, enabling continuous rAAV production. This study demonstrates the successful implementation of a continuous rAAV production process, yielding over 5 $\times$ 10^{10} vg/mL per day for 4 weeks. This work represents the first reported continuous rAAV production process utilizing the Sf9/BEVS platform and establishes an engineering strategy for overcoming manufacturing challenges associated with rAAV-based gene therapies.

INTRODUCTION

Recombinant adeno-associated virus (rAAV) has become the predominant vector for *in vivo* gene therapy due to its non-pathogenic nature, broad tissue tropism, and prolonged gene expression.[1–3] These promising attributes has paved the path to over 300 ongoing clinical trials using rAAVs for various conditions ranging from metabolic diseases to cancers.[4] Despite clinical success, vector production with a consistent quality at a scale suitable for widespread use remains a major challenge.[5] As of July 2025, five approved gene therapies utilize rAAVs packaged in HEK293 (mammalian) cells, while another three approved gene therapies rely on the insect cell/baculovirus expression system (IC/BEVS).[6] Given the extremely high dose requirements, which ranges from 1.5 $\times$ 10^{11} to 2 $\times$ 10^{11} viral genomes (vg) for localized administration, and up to 1.33 $\times$ 10^{14} vg/kg for systemic delivery,[7] there is a growing demand for a scalable and cost-effective vector production process.[8]

The state of the art of rAAV production is batch mode, either using IC/BEVS or triple-transient transfection of HEK293 cells. Although batch production is well established, continuous manufacturing represents a promising avenue for cost reduction and enhanced quality assurance.[9] For instance, certain small molecules and biologics can be manufactured using continuous production methods, which may offer several advantages: (1) high product titer, (2) reduced human intervention and risk of contamination, (3) low variability between harvests, and (4) lower cost.[10,11] Biologics manufactured using a continuous production mode include monoclonal antibodies, enzymes, and virus-like particles (VLPs).[12–15] For monoclonal antibodies, it was estimated that continuous production could decrease 68% of manufacturing costs in clinical trials and 35% for commercial use.[16] In addition, monoclonal antibodies produced using a Chinese hamster ovary cell line in a continuous run over 5 weeks were both consistent in quality and therapeutic efficacy.[12] Similarly, enzymes such as PNPGase have been produced consistently with continuous

Received 13 October 2025; accepted 24 April 2026;
https://doi.org/10.1016/j.omta.2026.201744.

[4]These authors contributed equally

Correspondence: Richard D. Braatz, Center for Biomedical Innovation, Massachusetts Institute of Technology, Cambridge, MA, USA.
E-mail: braatz@mit.edu
Correspondence: Stacy L. Springs, Center for Biomedical Innovation, Massachusetts Institute of Technology, Cambridge, MA, USA.
E-mail: ssprings@mit.edu

© 2026 The Author(s).
Published by Elsevier Inc. on behalf of The American Society of Gene and Cell Therapy.
This is an open access article under the CC BY license (http://creativecommons.org/licenses/by/4.0/).

runs of up to 40 days.[13] More recently, influenza VLPs were continuously generated through a production process using insect cells that lasted 20 days.[14,17] These previous studies indicate that continuous manufacturing could be a promising approach for IC/BEVS for rAAV production.

Production of rAAV in continuous mode is difficult to achieve using transient transfection in HEK293, producer cell lines, and IC/BEVS due to several factors that adversely affect yield, as discussed in the following texts. Although transient transfection is employed in large-scale batch production, its implementation in continuous mode is hampered by the large dose of vector plasmids required for repeated transfection and inefficient plasmid uptake on repeated transfection.[18,19] In addition, the preparation of fresh polyplex complexes and repeated dosing add to the cost and introduce the risk of contamination. Another bottleneck to continuous operation is the cytostatic and cytotoxic nature of nonstructural Rep proteins, leading to negative selection pressure, which may result in deletion of genes from stable producer cell lines.[20,21] The IC/BEVS is an attractive alternative to HEK293 cells due to the higher yield of full rAAV capsids that can be achieved.[22] Furthermore, the self-replication of recombinant baculovirus (rBV) in insect cells offers a natural avenue for cost reduction, as infected cells continuously produce progeny rBV. This eliminates the need for frequent manual intervention during vector production and reduces reliance on expensive GMP grade plasmid vectors required in HEK293-based systems.[23] However, a major issue associated with the rBV-based system is genetic instability, which complicates the maintenance of a consistent multiplicity of infection (MOI) of baculovirus with recombinant AAV genes.[24] Upon serial passaging, rBVs are prone to mutations that lead to the accumulation of two classes of defective viruses: (1) baculoviruses that have lost essential recombinant AAV genes, such as *rep*, *cap*, or the inverted-terminal-repeat-flanked gene of interest (GOI), and (2), defective interfering particles (DIPs) that lack critical genes required for baculovirus self-replication.[25] Both types of defective viruses disrupt the production of functional rAAV by impairing Rep and Cap protein expression, ultimately leading to a progressive decline and eventual halt of vector production.

In this study, we develop and quantify the first process for IC/BEVS-based continuous rAAV manufacturing that sustains high titers for 4 weeks. The process integrates a three-reactor cascade (growth, infection, and production) with controlled infusion of low-passage recombinant baculovirus to separate cell proliferation, primary infection, and late-stage baculovirus budding and rAAV production. Process design is guided by serial-passage characterization of baculovirus stability and a mechanistic model of baculovirus infection and propagation that predicts the process outcome for diverse operating conditions through *in silico* simulation.

RESULTS

Continuous rAAV production in the IC/BEVS fails due to rBV instability

A TwoBac system was used to evaluate continuous production of rAAV2/5 in insect cells (Figure 1A). The first type of rBV encodes the structural protein (cap5) and the AAV replication protein Rep2, while the second rBV carries the GOI flanked by inverted terminal repeat (ITR) elements. A reporter gene, enhanced green fluorescent protein (eGFP), was used as the GOI to monitor transduction efficiency.

A two-reactor cascade, consisting of a growth reactor and a production reactor (Figure 1B), was implemented to enable continuous rAAV production over 4 weeks. *Spodoptera frugiperda* (Sf9) cells were maintained at a density of 2.5–4 million cells/mL in the growth reactor throughout the process. A single inoculation of the TwoBac system at an MOI of 2 plaque-forming units (PFU) per cell in the production reactor (1 PFU per cell for each of the two recombinant baculoviruses) initiated rBV self-replication and rAAV production. After 48 h, uninfected Sf9 cells from the growth reactor were continuously transferred to the production reactor at a flow rate of 104 μL/min using a peristaltic pump. An average residence time of 60 h was maintained in both reactors to support cell replication in the growth reactor and rAAV production in the production reactor. Samples collected from the production reactor and a downstream harvest tank during the continuous run were analyzed to quantify total BV genome and infectious titer along with rAAV genome, and capsid titer (Figures 1C, 1D, and S1).

The initial rAAV genome titer exceeded 1×10^{11} vg/mL post 72 h rBV inoculation, but a sharp decline below 1×10^{10} vg/mL occurred after 12–15 days. By 22–25 days , the titer dropped below 1×10^{9} vg/mL, indicating that rAAV production had effectively reduced. Despite this decline, total rBV (measure by droplet digital polymerase chain reaction [ddPCR] using primers targeting late expression factor 2, *lef-2*) and infective BV titers remained relatively stable throughout the process (Figure 1C). These results suggest the accumulation of baculoviruses lacking the *rep* and/or *cap* cassettes are likely contributing to the observed loss of rAAV production.

Analysis of rBV genetic stability via serial passage

The decline in rAAV production observed in the two-tank cascade system prompted further investigation into the genetic stability of the rBV vectors used in the process. A serial passage of rBV encoding *rep* and *cap* gene cassettes was performed to investigate the dynamics of mutant rBV accumulation during vector production. Cells were infected in the first passage at MOI = 0.1 PFU/cell and, subsequently, sub-cultured every 72 h using a 1:100 fixed-volume transfer (Figure 2A). Primers were designed to target *lef-2* and rAAV gene (*rep* and *cap*) cassettes to quantify the total BV and rBV genome titers, respectively, using ddPCR. Loss of rAAV genes and protein expression was observed with increasing passage number of rBV (Figures 2B–2E). Deletion of the recombinant genes from rBV generates defective baculoviruses that interfere with rAAV production. Due to the shorter genome, the defective particles may potentially replicate and outcompete rBVs during continuous cultivation.

Process design for continuous rAAV production in the IC/BEVS

The experiments of continuous rAAV production in the two-tank cascade and BV serial-passage showed that rAAV productivity

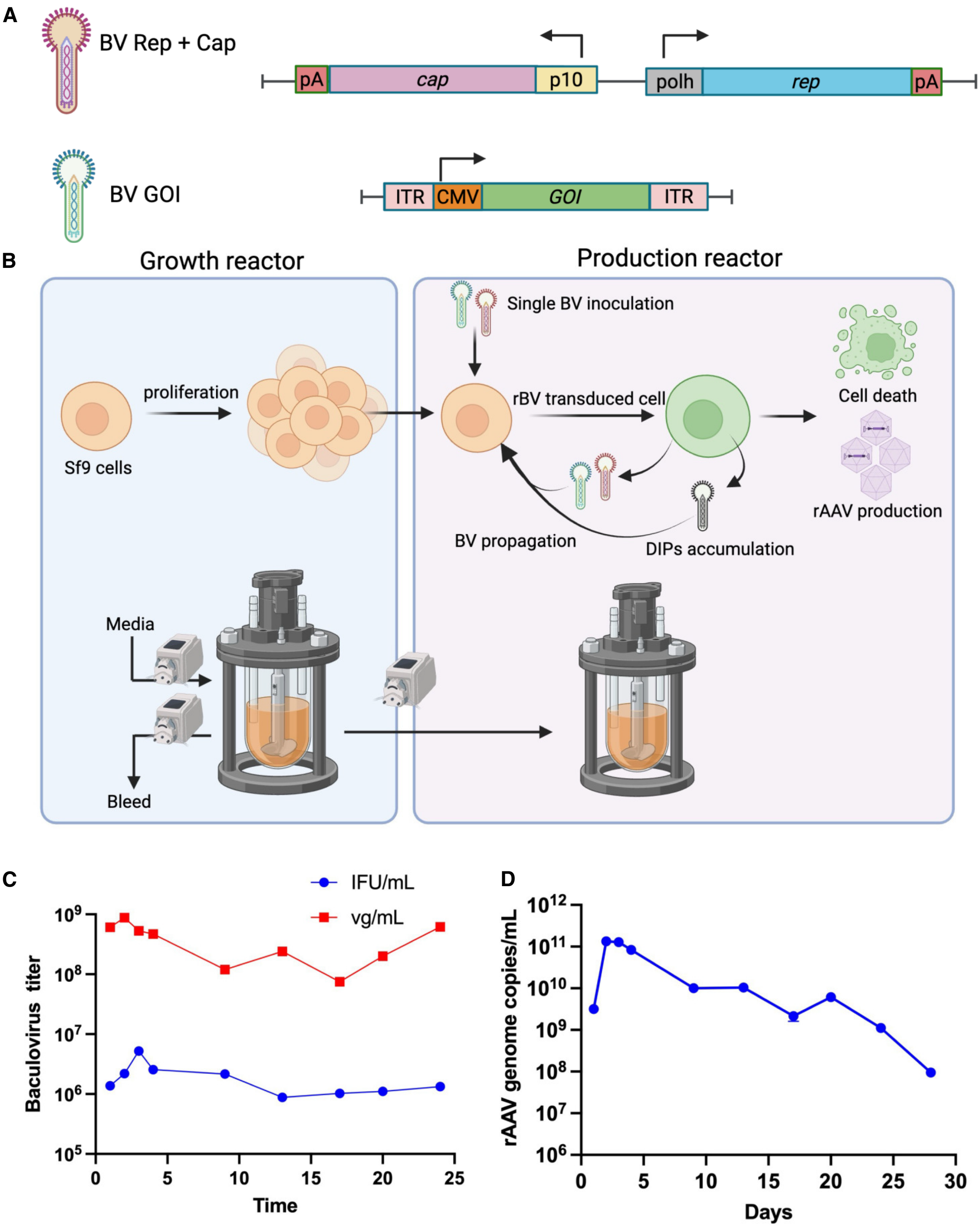

Figure 1. Continuous rAAV production in the IC/BEVS with a two-tank cascade

(A) TwoBac system encoding GOI and AAV *rep* and *cap* genes for packaging rAAV in Sf9 cells. (B) Two-reactor configuration with a residence time of 60 h per tank, facilitating the growth of Sf9 cells in the growth reactor and the self-propagation of rBV in the production reactor. (C) Budded baculovirus virions quantified from cell supernatant over 24 h. Total baculovirus genome titer and infectious forming units per mL. (D) Full rAAV titer quantified from cell suspension.

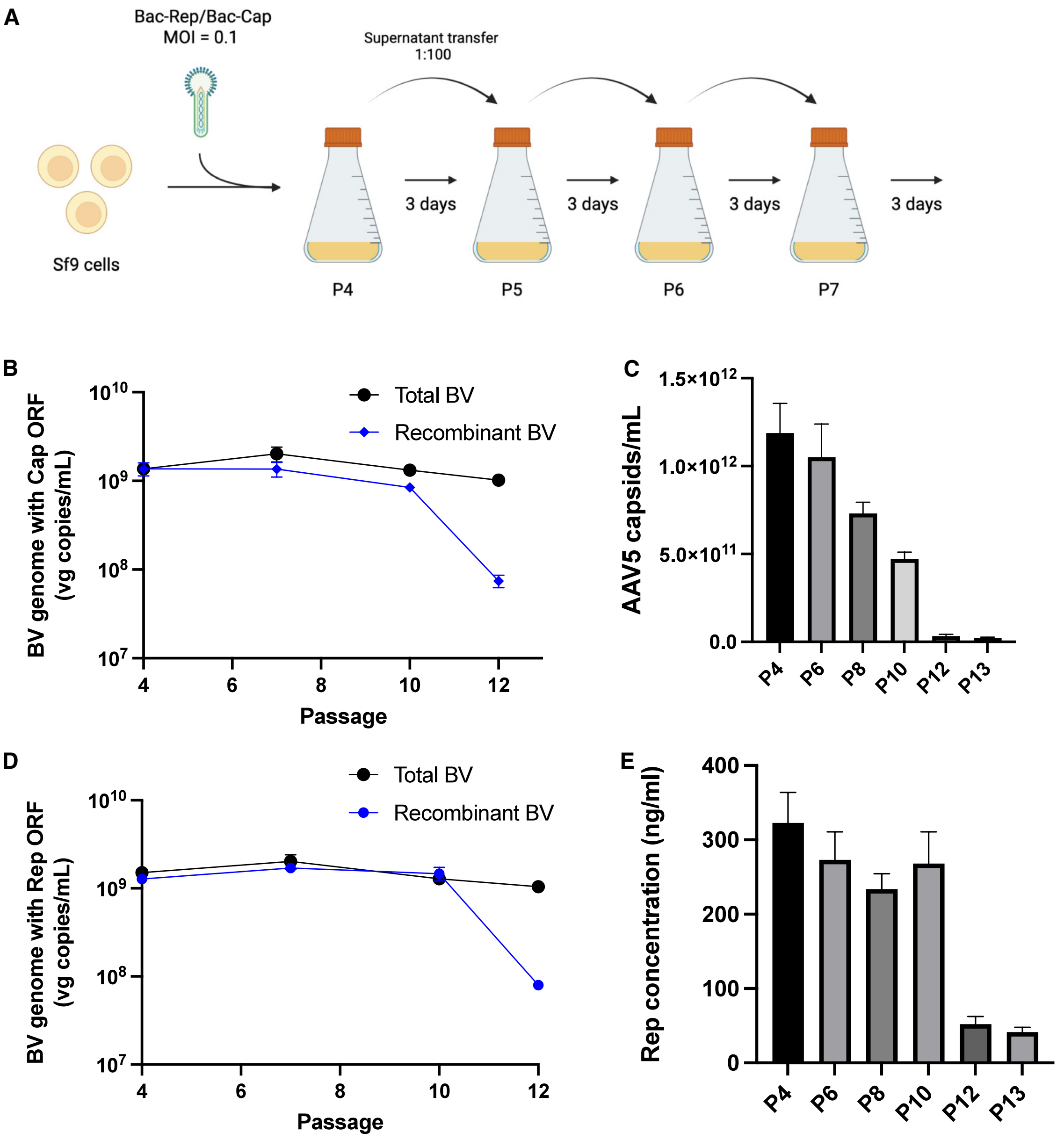

Figure 2. Analysis of genetic stability of rBV encoding rAAV genes through serial passages

(A) Experimental layout of serial passage for rBV in Sf9 cells. Cell culture supernatant containing the budded rBV is subcultured into a new flask containing uninfected Sf9 cells every 72 h. (B–E) Quantification of baculovirus and AAV proteins. (B) and (D) Total budded BV and rBV were quantified from cell supernatant using ddPCR. (C) and (E) Capsid and Rep proteins were quantified from cell suspension using ELISA.

declines when producer cells are predominantly infected by high-passage BVs lacking *rep* and/or *cap* cassettes. To mitigate this effect, a three-reactor cascade was designed, comprising growth, infection, and production bioreactors, with continuous cell transfer among reactors and continuous supplementation of low-passage (passage N = 4) rBV to the infection reactor (Figure 3A). This design aims at physically compartmentalizing (1) cell proliferation, (2) primary infection, and (3) baculovirus budding and rAAV production, leveraging the dependency of baculovirus kinetics on the amount of time that a cell has been infected (here referred to as *infection age*, measured in hours post-infection; hpi). Specifically, baculovirus reinfection is significantly reduced beyond 3–5 hpi due to virus-induced receptor downregulation.[26,27] In contrast, progeny baculovirus budding typically begins around 14–18 hpi and continues until cell lysis.[26,27]

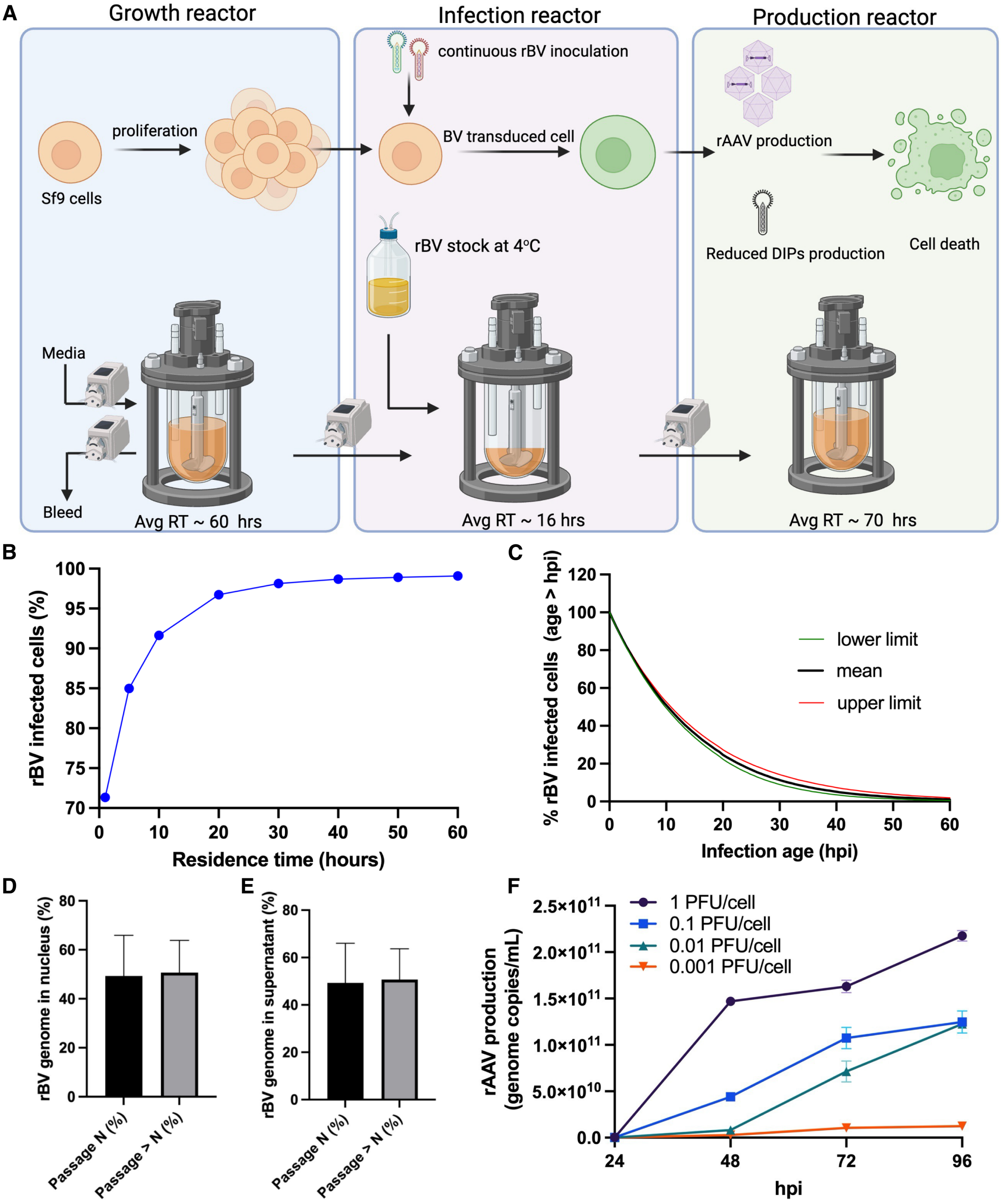

Figure 3. Process design for continuous rAAV production in the IC/BEVS with a three-tank cascade

(A) Scheme of three-reactor configuration. (B–E) Mechanistic model predictions for the infection reactor at steady state, with a feed of 2.5 million uninfected cells/mL and 2 PFU/cell: (B) Percentage of Sf9 cells infected with rBV as a function of residence time (RT). (C) Infection age distribution of rBV-infected Sf9 cells for residence time equal to 16 h. The *y* axis indicates the percentage of infected cells with infection age greater than the indicated hpi based on lower limit, mean, and upper limit estimates (D) and (E) passage *N* vs. passage greater than *N* rBV copy-number abundance in, respectively, the supernatant and nucleus of infected cells, for residence time equal to 16 h. The model predictions shown in (C)–(E) are the mean predictions across 200 Monte Carlo realizations, with 95% confidence limits reflecting uncertainty propagated from the joint parameter distribution. (F) rAAV production with the TwoBac platform at varying MOIs (0.001–1 PFU/cell per type of rBV) in a shake flask culture. The graph depicts the full rAAV genome copies determined using ddPCR from the cell lysate.

rAAV production is governed by very late promoters encoded in rBV that drive *rep* and *cap* expression, which are activated between 18 and 24 hpi.[28]

To implement this strategy effectively, a model-based process design was conducted to select appropriate values for the most critical operating conditions, namely the residence times of each reactor and the rBV concentration in the infection reactor feed. The objective was to ensure high infection rates from low-passage rBV in the infection reactor while minimizing reinfection in the production reactor, where defective baculoviruses accumulate. A mechanistic model of baculovirus infection and propagation in suspension cultures (materials and methods) supported the process design. The model enabled prediction of the infection-age distribution and the proportions of passage *N* and higher-passage baculoviruses in the infection reactor, both in the supernatant and within infected cells, under different operating conditions. Based on these simulations, a residence time of 16 h, a feed of 2.5 million uninfected cells/mL, and 2 PFU/cell per rBV type were selected to ensure more than 95% of the cells in the infection reactor are infected at steady state (Figures 3B and S2). Figure 3C depicts the model prediction of the steady-state infection age distribution of infected cells in these operating conditions. More than 80% of infected cells exceed 3 hpi of infection age, thereby minimizing reinfection in the downstream production reactor (Figure 3C). Only 30% of infected cells exceed 18 hpi (onset of BV progeny budding), while 20% and 3% exceed 24 and 48 hpi, respectively. Under these conditions, the model simulations also indicate that passage *N* rBV constitutes more than 50% of the BV genomes in the supernatant, with infected cells similarly containing more than 50% of their total nuclear BV genome derived from passage *N* (Figures 3D and 3E). In other words, the model estimates that approximately half of the rBV genome in the infection reactor originates from the feed viral passage rather than a later passage.

Additional simulations were conducted to evaluate alternative process designs that were ultimately deemed less favorable. An infection reactor with the same feed (2.5 million uninfected cells/mL and 2 PFU/cell per rBV type) but a higher residence time resulted in the predominance of high-passage BVs at steady state (Figure S2). For example, a 60-h residence time reduced the passage *N* genome fraction in infected cells and the supernatant to approximately 25% (Figure S3). This outcome underscores the advantage of a three-reactor system with a dedicated infection stage, which helps to maintain a high proportion of low-passage rBV in contrast to a simpler two-reactor configuration. Residence times shorter than 16 h were not explored, since they would increase the fraction of cells entering the production reactor at early infection ages (<3–5 h post-infection), when superinfection remains efficient, thereby favoring reinfection by defective baculoviruses. Further simulations showed that supplying 1 PFU/cell of each rBV type in the feed maintains approximately 45% passage *N* genomes in infected cells (Figure S4). These findings support the selection of 1–2 PFU/cell as an effective feed condition for preserving a low-passage rBV population during continuous operation.

While maintaining low-passage rBV is essential to minimize the accumulation of defective particles, rAAV production also requires that individual cells are coinfected by both baculovirus types (encoding *rep*, *cap*, or the ITR-flanked GOI). To evaluate the relationship between MOI and productive coinfection, shake flask experiments were conducted to evaluate rAAV production across a range of MOIs for rBV encoding *rep* and *cap* genes to determine the MOI required in the infection reactor to achieve coinfection from both types of rBV and high rAAV production (Figure 3F). The results indicated that rAAV production increases with MOI, reaching a peak at 2 PFU/cell, indicating that this level is sufficient to ensure coinfection in most cells at an initial seeding density of 1.5×10^6 cells/mL. These experimental findings were further supported by a previously developed mechanistic model for rAAV production in the IC/BEVS, which indicated that MOIs of 2 PFU/cell led to productive coinfection and high rAAV yields in approximately 75% and 90% of cells, respectively, in shake flask cultures.[29] In the continuous process, the effective MOI in the infection reactor reflects both the baculovirus feed and a (limited) amount of progeny rBV generated *in situ*, which partially retains functional *rep* and *cap* genes.

Based on the experimental and modeling results, a residence time of 16 h was selected for the infection reactor, with a target of 1.5–2 PFU/cell to simultaneously support productive coinfection and preserve a sufficient proportion of low-passage rBV. Residence times of 60 and 70 h were chosen for the growth and production reactors, respectively, to allow sufficient time for cell proliferation and rAAV production, consistent with Sf9 growth and rAAV expression kinetics in the IC/BEVS.

Continuous, stable rAAV production in a three-tank cascade with continuous rBV feed

Continuous rAAV production in a three-tank cascade was implemented based on the process design described in the previous section (Figure 3A). Sf9 cells were maintained at a cell density of 2.5–4 million cells/mL in the three reactors during production. A constant feed of rBV was supplied via a peristaltic pump into the infection reactor, ensuring approximately 1.5–2 PFU/cell per type of rBV in the overall feed to the infection reactor. Samples from the infection reactor were collected to identify defective baculoviruses using ddPCR. To this end, we designed primers to target the *rep* and *cap* genes and *lef-2*. Based on the ddPCR readout, it is evident that all three primers showed similar gene amplification, which is indicative of *rep* and *cap* sequence conservation in the continuous production process (Figure 4A). The rAAV genome and capsid titers were quantified from the infection reactor, production reactor, and harvest during the continuous run for 28 days using ddPCR and ELISA (Figures 4B and 4C). The rAAV genome and capsid titers showed stable production over the 28-day production period (Figures 4B and 4C). Total rep proteins quantified from cells lysate shows stable expression during the production period (Figure 4D). SYPRO Ruby staining of denatured rAAV capsids produced from the continuous production was performed to analyze VP1, VP2, and VP3 proteins (Figure 4E).

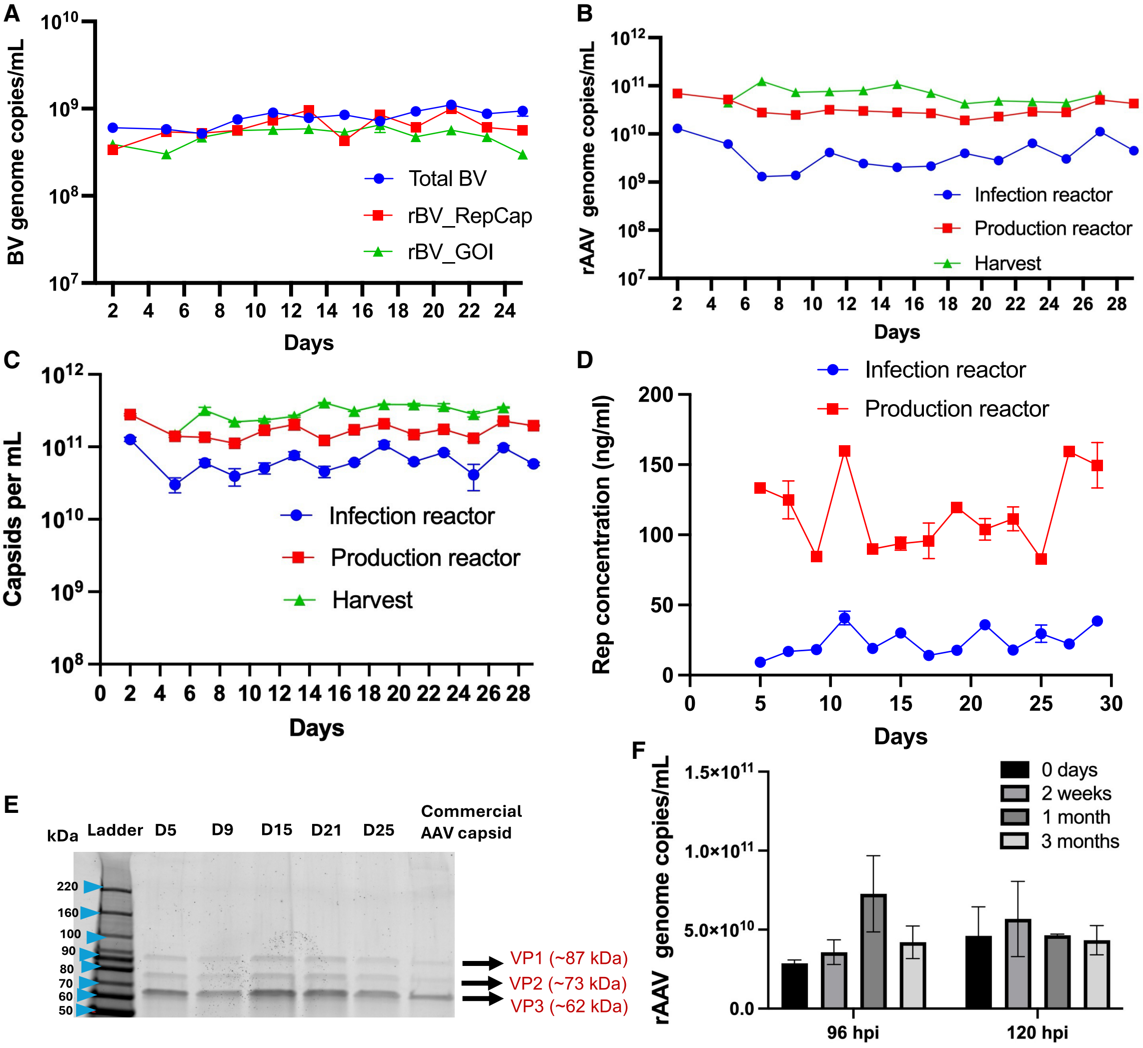

Figure 4. Continuous rAAV production using a three-tank cascade

(A) Total BVs and rBVs were quantified from cell culture supernatant using ddPCR with primers to target the *lef2* and AAV genes (B) rAAV genome titer was quantified from cell suspension quantified using ddPCR. (C) Quantification of total AAV capsid produced during the continuous production from cell suspension (D) Rep concentration from cell lysates using ELISA (E) SYPRO Ruby staining of denatured AAV capsids produced from the continuous production showing viral proteins (VPs). (F) Infectivity of budded rBV stored in spent media for 3 months at 4°C. The budded rBVs collected at different time points were used to infect Sf9 cells to produce rAAV.

The shelf stability of rBVs in crude spent media stored at 4°C was used for the rAAV production and showed no statistically significant difference (Figure 4F). Here, rAAV titer was quantified to measure infectivity of rBVs as is relevant in the context of continuous AAV production. Overall, the experiment yielded 4.5 × 10^{14} full rAAV capsids across 28 days, representing approximately 300% increase compared to the two-tank system control shown in Figure 1. Compared to batch-mode production over the same 28-day period, the process achieved an 80% increase in total rAAV output. This comparison assumes the same three bioreactors are used in batch mode for 4 weeks, with one batch per run per week and a final full rAAV titer of 1 × 10^{11} vg/mL at the end of each batch. The overall continuous production process resulted in a full capsid percentage of 20.5 ± 6.5, which was comparable to batch run with an average filling of 26.6 ± 3.5% (Figures S5 and S6). The transduction potential of rAAV from the continuous production was evaluated in HEK293 cells. GFP expressions were comparable throughout the continuous production period and were also similar to batch run, as shown in Figures S7 and S8.

An additional control experiment was carried out in the three-tank system with a 60-h residence time for each reactor and no continuous rBV feed to the infection reactor (Figure S9). Sharp declines in the rAAV genome and capsid titer were registered 10 days after the process onset (Figures S9A and S9B). The rAAV genome titer decrease correlates with the reduced Rep protein expression

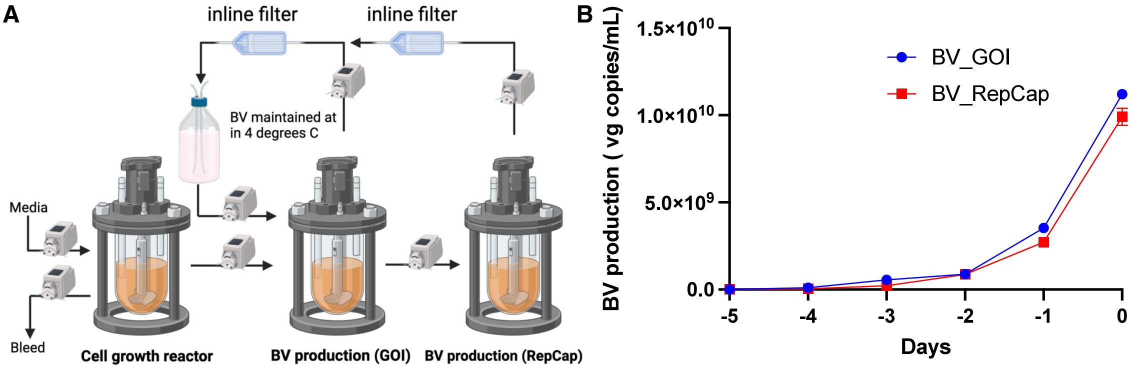

Figure 5. Procedure for generation of the recombinant baculovirus stock for continuous rAAV production

(A) Schematics of the rBV generation workflow. This involves the expansion of rBV from a single inoculation, followed by the harvest of budded rBV using inline filters. The two types of rBVs were quantified using ddPCR and combined to make the stock at a 1:1 ratio before storage at 4°C. (B) Production kinetics and rBV volumetric yield of the budded virions during the stage 1 expansion step.

(Figure S9C). In the next section, a fast and efficient approach that was employed to generate the baculovirus stock is described.

Fast, efficient generation of the baculovirus stock for continuous rBV feed

An efficient expansion step is required for producing a sizable viral stock to sustain continuous infusion of both types of rBV for 4 weeks. We developed an approach to expand the rBV stock and maintain rBVs at a low passage number for several weeks. In our study, 20 μL of rBV stock preserved as baculovirus-infected insect cells (BIICs) was scaled up to 400 mL in 5 days. rBVs were harvested using inline filters (1 micron pore size) after 5 days of infection with rBV at MOI = 0.0001 PFU/cell. ddPCR was performed to quantify rBV, and the ratio of both types of rBV was adjusted to 1:1 before storage at 4°C (Figure 5A). The production kinetics of rBV from the bioreactors revealed a rBV yield above 1×10^{10} vg/mL (Figure 5B). The rAAV production from budded rBV at different titers was compared to the rAAV production method using BIICs, as demonstrated in Figure S10.

DISCUSSION

Continuous rAAV production in the IC/BEVS is a promising strategy for reducing costs of goods sold and increasing volumetric productivity compared to traditional batch production. However, a significant challenge in implementing continuous production in the IC/BEVS is the formation and propagation of defective baculoviruses due to deletion events during rBV propagation.[30] Our first attempt of continuous rAAV production in the IC/BEVS, employing a two-reactor cascade, was unsuccessful, with rAAV yields declining significantly after 1–2 weeks of continuous operation (Figure 1).

Our serial passage experiments provided clear evidence of an increase in defective baculovirus populations lacking *rep* and/or *cap* genes, which became predominant beyond passage 12 (Figure 2). These observations highlighted the necessity of maintaining low-passage rBV populations to ensure genetic stability and sustained rAAV production. While previous studies demonstrated improved genetic stability using FlashBac compared to Bac-to-Bac systems to generate the rBV stock,[14,31,32] the long term genetic stability of FlashBac is yet to be investigated.

Here, we introduced a novel process for rapid, efficient transition of batch-optimized rBVs to continuous production without the need for baculovirus redesign. Our process comprises a continuous three-tank cascade of growth, infection, and production reactors, with continuous infusion of low-passage rBV stocks into the infection reactor (Figure 3A). The physical separation of infection stages provides control over infection dynamics, improving Sf9 cell replication, baculovirus propagation, and ultimately, rAAV production. Crucially, defective baculoviruses accumulate primarily in the production bioreactor, where cells arrive at late infection stages and are refractory to reinfection. A mechanistic model of baculovirus infection and propagation supported the process design, enabling *in silico* simulations that evaluated infection efficiency and infection age and baculovirus passage distributions in the infection reactor, under varying residence times and rBV feed concentrations (Figures 3B–3E and S2–S4). Our experimental implementation validated these predictions, successfully demonstrating continuous rAAV production over a prolonged 28-day period.

We also developed a novel strategy for generating and maintaining low-passage rBV stocks throughout the whole duration of the continuous process. For continuous feed of rBV to the infection reactor and long-term storage of rBV it was essential to use budded rBV rather than BIICs. Notably, shelf stability studies demonstrated that rBV stocks retained their infectivity and showed no significant degradation after storage at 4°C for 3 months. This robustness enables practical storage flexibility, allowing the generation of the entire rBV stock several days before initiating continuous production without compromising productivity.

A critical aspect of continuous rAAV production that necessitates further investigation is the integrity of the ITR. These *cis*-acting elements are essential for rescue and amplification, genome encapsidation, and long-term transgene expression.[33] However, ITRs are susceptible to truncations and mutations due to high GC content and palindromic sequences.[34] Moreover, truncated or mutated ITRs in the Sf9/BEVS system resulted in a lower fraction of full capsids compared to constructs with wild-type ITRs.[35] The risk of ITR instability may become amplified during continuous production with each BV replication cycle. Our study utilizes low-passage baculoviruses to mitigate this effect; however, long and short-read sequencing are needed to assess ITR integrity and foreign DNA impurities such as baculovirus and host cell genome. Another study limitation that remains unexplored includes the effect of AAV serotypes and transgene cargo size on continuous production. This is an important aspect as the quality attributes including product yield and full capsids can change due to differences in the kinetics of capsid assembly and genome packaging capacity.[36]

This study represents the first reported continuous rAAV production utilizing the IC/BEVS, effectively addressing critical manufacturing challenges related to defective baculovirus formation and genetic stability. From a pharmaceutical manufacturing standpoint, the proposed three-reactor cascade is compatible with standard stirred-tank bioreactors and existing continuous upstream infrastructure. The main additional requirements are a low passage rBV seed train and a chilled hold vessel to supply the infection reactor, without introducing new or specialized unit operations. Our simulations and experiments indicate that optimization efforts should focus on reactor residence times, feed cell concentration and rBV MOI, and media composition, which jointly govern both the defective rBV fraction and rAAV productivity. Together with our 28-day stable operation and prior reports of multi-stage BEVS cascades,[14] these results support the practical feasibility and scalability of this strategy for continuous rAAV manufacturing. Moreover, the strategies and insights developed here can be broadly extended to other continuous viral manufacturing systems similarly challenged by defective virus formation.

MATERIALS AND METHODS

Cell culture experiments, viral transduction, and quantification of viral titers were performed following BL2 procedures approved by MIT's Environmental, Health, and Safety (EHS) committee.

Cell lines and culture media

Sf9 suspension cells were maintained at a cell density ranging from 0.3 to 3 × 10^6 cells/mL in serum-free SFM4 media (HyClone, Utah, USA). The Sf9 cells were cultured in a sterile 125-mL Erlenmeyer flask (Fisher Scientific brand sterile PC flasks, Cat. #PBV125). These cells were cultured at 27°C in an incubator (Thermo Scientific HERA cell VIOS 160i) on an orbital shaker (ORBI SHAKER CO2, Benchmark) at 135 rpm.

Serial passaging of baculovirus

Sf9 cells, at a cell density of 1.5 × 10^6 cells/mL, were added to a 125 mL Erlenmeyer flask and inoculated with recombinant BV-encoding *rep* and *cap* genes at an MOI = 0.1 PFU/cell. Supernatant was harvested every 3 days and subsequently inoculated into a new flask of uninfected Sf9 cells using a 1:100 fixed-volume transfer from passages 4 to 12. Samples collected from passages 4, 7, 10, and 12 were used for ddPCR after digestion with a DNAse I (New England Biolabs, MA, USA).

Baculovirus infectious titer determination

Recombinant baculovirus infectious titers were quantified using the BacPAK Baculovirus Rapid Titer Kit (Takara Bio, Kyoto, Japan) according to the manufacturer's protocol. Briefly, Sf9 cells were seeded in 96-well microtiter plates not exceeding 6.5 × 10^4 cells/well. After 1 h of incubation, the adherent Sf9 cells were inoculated with 10-fold serial dilutions of baculovirus stocks. An hour of incubation was provided to facilitate virus attachment and entry. After the removal of virus inoculum and wash, cells were overlaid with methylcellulose and incubated at 27°C for 45 h. Infected cells were fixed, then immunostained using a monoclonal gp64-antibody raised in mice. An HRP-conjugated anti-mouse antibody and substrate development were used to stain the infected cell. Infectious foci were counted by light microscopy, and viral titers were calculated as infectious-forming units per milliliter (IFU/mL). A conversion factor provided by the manufacturer was used to convert IFU to PFU (1 IFU/mL = 2 PFU/mL).

Quantification of vector genome

ddPCR reaction mix was prepared with EvaGreen Supermix following the manufacturer's protocol. Samples were diluted in DNase/RNase-Free Deionized Water (Thermo Fisher Scientific, MA, USA). Droplet generation was performed using Droplet Generation Oil for EvaGreen on the QX200 Automated Droplet Generator. Thermal cycling was performed with the C100 Touch Thermal Cycler using a heated lid at 105°C and a sample volume of 40 μL and a protocol as follows: enzyme activation at 95°C for 5 min, 40 cycles of denaturation at 95°C for 30 s, annealing and extension at 60°C for 1 min, signal stabilization at 4°C for 5 min, and signal stabilization at 90°C for 5 min. Droplet reading was done with the QX200 Droplet Reader (Bio-Rad, MA, USA), and all plate sealing was done with the PX1 PCR plate sealer.

Quantification of recombinant and total baculovirus genome titer

Baculovirus titer budded from infected cells was quantified from the spent media using primers that target the AAV gene (*rep* and *cap*) cassettes and a conserved baculovirus gene (*lef-2*). The primer sequences are:

Rep Forward: 5′-GCAGACAATGCGAGAGAATG-3'

Rep Reverse: 5′-CACGGGAAAGCACTCTAAAC-3'

Cap Forward: 5′-CATCGGCACCTTGGTTATTG-3’

Cap Reverse: 5′-CTACCGGAAAGCGGATAGAC-3’

lef-2 Forward: 5′-GAAGAAGCTGCGTAGTATGCC-3’

lef-2 Reverse: 5′-GTAGTTCTTCGGAGTGTGTTGC-3’

Quantification of AAV genome titer

Cells were lysed with 1× lysis buffer (20% Tris HCl, 1% MgCl2, 5% Tween 20, and 74% Milli-Q Water) and then centrifuged for 5 min at 10,000 rpm to remove cell debris. Host genomic DNA, baculovirus genome, and unpackaged AAV genome were digested with 10U DNase I (New England Biolabs, Massachusetts, USA) for 1 h and heat-inactivated at 75°C for 10 min. ddPCR was performed as described above using eGFP primers with the following sequences:

eGFP Forward: 5′-GCAAAGACCCCAACGAGAAG-3’.

eGFP Reverse: 5′-TCACGAACTCCAGCAGGACC-3’.

Quantification of total rAAV capsids

Cells were lysed with 1× lysis buffer for 1 h and then were centrifuged for 5 min at 10,000 rpm to remove cell debris. AAV5 Xpress ELISA kits (PROGEN, Pennsylvania, USA), were used as per the manufacturer’s protocol. Optical density (OD) was measured via a BioTek Synergy H1 Plate Reader (Agilent Technologies, Vermont, USA). Unknown concentrations were interpolated via a 4-parameter logistic fit (4PL) for capsid titer ELISAs.

Determination of total rep proteins

Cell lysates were used for the quantification of total Rep proteins using AAV Rep ELISA kit (Cell Biolabs, California, USA) according to the manufacturer’s protocol. Unknown concentrations were interpolated with a linear standard curve.

Influence of MOI on rAAV production

Sf9 cells were seeded at a density of 1.5×10^6 cells/mL in Erlenmeyer flasks containing 25 mL of SFM4 culture media. Cells were inoculated with the TwoBac platform, which consists of rBV encoding the AAV rep and cap genes and rBV encoding GFP flanked by ITRs. The MOI of rBV was varied at 0.002, 0.02, 0.2, and 2 PFU/cell. Cultures were incubated at 27°C in an orbital shaker at 135 rpm. At 72 h post-infection, cells were lysed and treated with DNase I as described previously. The rAAV genome titers were quantified by ddPCR. All conditions were performed in triplicate.

Continuous AAV production

Sf9 cells were inoculated in a stirred-tank bioreactor (OmniBRx Biotechnologies, USA and Applikon, Holland, the Netherlands) connected by peristaltic pumps that allowed for continuous production. All three reactors (vessel volume 500 mL) were inoculated at 0.5×10^6 cells/mL in 300 mL (unless specified) SFM4 Insect media (HyClone, Utah, USA). Each reactor was held at 27°C, a pH of 6.1–6.5, with 70% dissolved oxygen maintained using a microsparger. Reactors were also equipped with three-blade marine impellers rotating at 130 rpm. The media stock was protected from light and maintained at 4°C. The cell density in the growth reactor was maintained between 2–4 $\times 10^6$ cells/mL. A controlled bleeding was implemented from the growth reactor when the cell density exceeded 4×10^6 cells/mL. Cells were added to growth reactor if the cell density fell below 2×10^6 cells/mL. All infections were performed using a dual baculovirus system encoding the Rep/Cap proteins and the GOI.

Single baculovirus inoculation

Baculovirus was introduced into the infection reactor at an MOI = 1–2 PFU/cell when the Sf9 cells reached a target density of 2–3 $\times 10^6$ cells/mL. The bioreactor was subsequently operated in batch mode for 48 h, after which continuous production was initiated by activating the peristaltic pumps at a flow rate of 85 μL/min. Cells in the growth reactor were maintained at a concentration below 4×10^6 cells/mL through controlled bleeding. Cells were replenished when the cell density fell below 2×10^6 cells/mL. Samples were collected every other day from all reactors to be used for determining AAV and capsid titers.

Continuous baculovirus inoculation

Continuous AAV production by BV inoculation strategy was done in 2 stages.

Baculovirus expansion in bioreactor

When SF9 cells in Reactor 2 and Reactor 3 reached 1.5×10^6 cells/mL, both were inoculated with baculovirus at an MOI = 0.001 PFU/cell. GOI baculovirus was introduced into Reactor 2, while Rep/Cap baculovirus was introduced into Reactor 3. After infection, inline filters (HyClone) were used to remove cellular debris from the cultures, and the clarified filtrate was pumped into a collection container maintained at 4°C.

Continuous rBV feed

The clarified baculovirus-containing filtrate was continuously pumped into Reactor 2 to maintain an MOI of 2 PFU/cell. After 24 h, continuous AAV production was initiated by activating the peristaltic pumps, allowing for steady-state infection and capsid production.

Quantification of VP proteins ratio

Sf9 cell suspension was first lysed as detailed previously, and cell lysate was purified using Dynabeads CaptureSelect AAVX Magnetic Beads (Thermo Scientific, Massachusetts, USA), as per the manufacturer’s protocol. Purified capsid samples were mixed with Laemmli SDS sample buffer, reducing (6×) (Thermo Scientific, Massachusetts, USA) and placed upon a heat block at 90°C. After 20 min, samples, along with BenchMark Protein Ladder (Invitrogen, Massachusetts, USA), were loaded onto a gel, and sodium dodecyl sulfate-polyacrylamide gel electrophoresis (SDS-PAGE) was performed. 4%–15% Mini-PROTEAN TGX Precast Protein Gels (Bio-Rad, Massachusetts, USA) and 1× Tris/Glycine/SDS (Bio-Rad) were used for

the electrophoresis. Gel was then stained with SYPRO Ruby Protein Gel Stain (Invitrogen, Massachusetts, USA) according to the manufacturer's protocol. Images were taken using a ChemiDoc MP Imaging System (Bio-Rad).

rAAV transduction efficacy

HEK293 cells were plated in a 24-well plate at a density of 0.7 million cells per well. Transduction was performed at 2,500 vg/cell and the transduced cells were imaged for GFP expression after 72 h. Commercial rAAV vectors produced by transient transfection were used as a reference and compared against rAAV vectors generated by continuous and batch production using Sf9/BEVS. Mean fluorescence intensity were quantified using ImageJ.

Long-term stability of recombinant baculovirus

Sf9 cells cultured at 1.5×10^6 cells/mL were inoculated with BV infected cells (BIICs-GOI and Rep-Cap, separately) at a dilution level of 1/10,000. The BIICs were preserved in a freezing media consisting of 10% DMSO and 150 mM Trehalose in SFM4 Insect media (HyClone, Utah, USA). The newly infected cells were checked daily until viability dropped below 80% and average cell diameter increased by 3–4 μL, indicating infection. The supernatant was collected after spinning down at 10,000 RPM for 5 min, was collected and stored at 4°C. To quantify infectivity of rBV, samples containing budded rBVs were added to media containing uninfected Sf9 cells (1.5×10^6 cells/mL) at an MOI = 1 PFU/cell. After 96 h, the cells were harvested and lysed. Cell lysis was performed as described previously, and cell lysate was assayed for copies of AAV genomes using ddPCR.

Mechanistic model of baculovirus infection and propagation

The mechanistic model of baculovirus infection and propagation is adapted from Destro and Braatz, where a more detailed description of the model is provided.[37] The model describes viral infection and propagation in a well-mixed tank, operated in batch or continuous mode. The model accounts for the presence of two viral species in the system: baculovirus of passage N (virus 1) and baculovirus of passage greater than N (virus 2). Based on the experimental conditions followed in this work, $N = 4$ was set. The full set of model equations is reported in the supplemental information (Equations S1–S23). Briefly, the model inputs are the initial conditions for all the system states (Table S1), the reactor operating mode (batch or continuous) and, for continuous operating mode, the residence time and the feed concentrations of viable and nonviable uninfected cells and virions of type 1 and 2. The model outputs are the time evolution profiles of all the system states, namely the concentration within the reactor volume of uninfected viable cells, viable cells infected only by virus 1 or 2, viable cells coinfected by virus 1 and virus 2, nonviable cells, free virions of type 1 and 2, virus 1 bound to viable cells infected only by virus 1, virus 2 bound to viable cells infected only by virus 2, virus 1 and virus 2 bound to viable coinfected cells, virus 1 in nucleus of viable cells infected only by virus 1, virus 2 in nucleus of viable cells infected only by virus 2, virus 1 and virus 2 in nucleus of viable coinfected cells. All concentrations of infected cells, cell-bound virus, and nuclear viral genomes are dynamically tracked over time and resolved by the relevant infection age(s): for cells infected by only virus 1 or only virus 2, the model tracks the corresponding infection age; for coinfected cells, both infection ages are tracked independently. The main viral infection and propagation steps captured by the model are: baculovirus infection, trafficking to nucleus, replication, and viral progeny production. The model explicitly accounts for the dependence of re-infection, intracellular replication, infected cell death, and virion release kinetics on the infection age of each cell. To generate the results presented in this manuscript, model inputs were specified according to the conditions of each experiment. The model equations (Equations S1–S23) were solved using a numerical method described previously.[37] All parameter values used in the simulations are listed in Table S2. These parameters, along with their confidence intervals, were originally estimated in Destro et al.[29] based on a combination of published data and in-house experiments on baculovirus infection and propagation kinetics. The only parameter re-estimated for this study was the baculovirus progeny production rate, which was inferred via maximum likelihood estimation using data from the serial passage experiments. Uncertainty was propagated into the model predictions using 200 Monte Carlo realizations, each sampling a parameter set independently from the joint parameter distribution.

DATA AND CODE AVAILABILITY

The data supporting the findings of this study are available upon request.

ACKNOWLEDGMENTS

The research was supported by the U.S. Food and Drug Administration under contract no. 75F40121C00131. The Sf9 cells and recombinant baculoviruses encoding AAV genes were generously gifted by Robert Kotin, University of Massachusetts, USA. The authors gratefully acknowledge OmniBRx Biotechnologies, specifically Ravindra Patel, Anandprakash Joshi, and Ravikumar Daraji for offering the bioreactors and technical support.

AUTHOR CONTRIBUTIONS

J.J., conceptualization, methodology, formal analysis, investigation, data curation, writing – original draft, writing – review and editing, and visualization; F.D., conceptualization, methodology, software, model validation, formal analysis, investigation, data curation, writing – original draft, and writing – review and editing; A.Y., D.A., W.C., S.S., M.T., C.Y., A.S., C.L., and T.B., methodology, investigation, data curation, formal analysis, investigation, and writing – review and editing; H.A.H., P.M., J.X.F., A.R.R., S.S., and C.C., data curation and investigation; C.N., funding acquisition; J.M.W., formal analysis, resources, data curation, writing – review and editing, and funding acquisition; P.S., conceptualization, methodology, formal analysis, visualization, and investigation; P.W.B., conceptualization, methodology, formal analysis, resources, data curation, writing – original draft, writing – review and editing, and funding acquisition; A.J.S., resources, data curation, writing – original draft, writing – review and editing, supervision, and funding acquisition; R.D.B., conceptualization, methodology, resources, data curation, writing – original draft, writing – review and editing, and funding acquisition; S.L.S., conceptualization, methodology, resources, data curation, resources, writing – original draft, writing – review and editing, and funding acquisition. All authors have reviewed and approved the final version of the manuscript.

DECLARATION OF INTERESTS

The authors declare no conflict of interest.

SUPPLEMENTAL INFORMATION

Supplemental information can be found online at https://doi.org/10.1016/j.omta.2026.201744.

REFERENCES

1. Niemeyer, G.P., Herzog, R.W., Mount, J., Arruda, V.R., Tillson, D.M., Hathcock, J., van Ginkel, F.W., High, K.A., and Lothrop, C.D., Jr. (2009). Long-term correction of inhibitor-prone hemophilia B dogs treated with liver-directed AAV2-mediated factor IX gene therapy. Blood *113*, 797–806. https://doi.org/10.1182/blood-2008-10-181479.
2. Walkey, C.J., Snow, K.J., Bulcha, J., Cox, A.R., Martinez, A.E., Ljungberg, M.C., Lanza, D.G., De Giorgi, M., Chuecos, M.A., Alves-Bezerra, M., et al. (2025). A comprehensive atlas of AAV tropism in the mouse. Mol. Ther. *33*, 1282–1299. https://doi.org/10.1016/j.ymthe.2025.01.041.
3. Herzog, R.W., Hagstrom, J.N., Kung, S.-H., Tai, S.J., Wilson, J.M., Fisher, K.J., and High, K.A. (1997). Stable gene transfer and expression of human blood coagulation factor IX after intramuscular injection of recombinant adeno-associated virus. Proc. Natl. Acad. Sci. USA *94*, 5804–5809. https://doi.org/10.1073/pnas.94.11.5804.
4. Srivastava, A. (2023). Rationale and strategies for the development of safe and effective optimized AAV vectors for human gene therapy. Mol. Ther. Nucleic Acids *32*, 949–959. https://doi.org/10.1016/j.omtn.2023.05.014.
5. Jiang, Z., and Dalby, P.A. (2023). Challenges in scaling up AAV-based gene therapy manufacturing. Trends Biotechnol. *41*, 1268–1281. https://doi.org/10.1016/j.tibtech.2023.04.002.
6. A Reid, C., Hörer, M., and A Mandegar, M. (2024). Advancing AAV production with high-throughput screening and transcriptomics. Cell Gene Therapy Insights *10*, 821–840. https://doi.org/10.18609/CGTI.2024.095.
7. Vrellaku, B., Sethw Hassan, I., Howitt, R., Webster, C.P., Harriss, E., McBlane, F., Betts, C., Schettini, J., Lion, M., Mindur, J.E., et al. (2024). A systematic review of immunosuppressive protocols used in AAV gene therapy for monogenic disorders. Mol. Ther. *32*, 3220–3259. https://doi.org/10.1016/j.ymthe.2024.07.016.
8. Collins, L.T., Ponnazhagan, S., and Curiel, D.T. (2023). Synthetic Biology Design as a Paradigm Shift toward Manufacturing Affordable Adeno-Associated Virus Gene Therapies. ACS Synth. Biol. *12*, 17–26. https://doi.org/10.1021/acssynbio.2c00589.
9. Kumar, A., Udugama, I.A., Gargalo, C.L., and Gernaey, K.V. (2020). Why Is Batch Processing Still Dominating the Biologics Landscape? Towards an Integrated Continuous Bioprocessing Alternative. Processes *8*, 1641. https://doi.org/10.3390/pr8121641.
10. Zydney, A.L. (2016). Continuous downstream processing for high value biological products: A Review. Biotechnol. Bioeng. *113*, 465–475. https://doi.org/10.1002/bit.25695.
11. Wahlich, J. (2021). Review: Continuous Manufacturing of Small Molecule Solid Oral Dosage Forms. Pharmaceutics *13*, 1311. https://doi.org/10.3390/pharmaceutics13081311.
12. Zhu, H., and Yang, S.-T. (2004). Long-term Continuous Production of Monoclonal Antibody by Hybridoma Cells Immobilized in a Fibrous-Bed Bioreactor. Cytotechnology *44*, 1–14. https://doi.org/10.1023/b:cyto.0000043395.36188.bc.
13. Gelain, L., Kingma, E., Geraldo da Cruz Pradella, J., Carvalho da Costa, A., van der Wielen, L., and van Gulik, W.M. (2021). Continuous production of enzymes under carbon-limited conditions by *Trichoderma harzianum* P49P11. Fungal Biol. *125*, 177–183. https://doi.org/10.1016/j.funbio.2020.10.008.
14. Correia, R., Zotler, T., Ferraz, F., Fernandes, B., Graça, M., Pijlman, G.P., Alves, P.M., and Roldão, A. (2025). Continuous Production of Influenza VLPs Using IC-BEVS and Multi-Stage Bioreactors. Biotechnol. Bioeng. *122*, 846–857. https://doi.org/10.1002/bit.28925.
15. Leong, J., Tang, W.Q., Chng, J., Ler, W.X., Manan, N.A., Sim, L.C., Zheng, Z.Y., Zhang, W., Walsh, I., Zijlstra, G., et al. (2024). Biomass specific perfusion rate as a control lever for the continuous manufacturing of biosimilar monoclonal antibodies from CHO cell cultures. Biotechnol. J. *19*, 2400092. https://doi.org/10.1002/biot.202400092.
16. Gupta, P., Kateja, N., Mishra, S., Kaur, H., and Rathore, A.S. (2021). Economic assessment of continuous processing for manufacturing of biotherapeutics. Biotechnol. Prog. *37*, e3108. https://doi.org/10.1002/btpr.3108.
17. Matsuda, T., Tanijima, T., Hirose, A., Masumi-Koizumi, K., Katsuda, T., and Yamaji, H. (2020). Production of influenza virus-like particles using recombinant insect cells. Biochem. Eng. J. *163*, 107757. https://doi.org/10.1016/j.bej.2020.107757.
18. Srinivasan, P., Canova, C.T., Sha, S., Nguyen, T.N.T., Joseph, J., Sangerman, J., Maloney, A.J., Katsikis, G., Ou, R.W., Hong, M.S., et al. (2024). Multidose transient transfection of human embryonic kidney 293 cells modulates recombinant adeno-associated virus2/5 Rep protein expression and influences the enrichment fraction of filled capsids. Biotechnol. Bioeng. *121*, 3694–3714. https://doi.org/10.1002/bit.28828.
19. Liu, S., Li, J., Peraramelli, S., Luo, N., Chen, A., Dai, M., Liu, F., Yu, Y., Leib, R.D., Li, Y., et al. (2024). Systematic comparison of rAAV vectors manufactured using large-scale suspension cultures of Sf9 and HEK293 cells. Mol. Ther. *32*, 74–83. https://doi.org/10.1016/j.ymthe.2023.11.022.
20. Pereira, D.J., McCarty, D.M., and Muzyczka, N. (1997). The adeno-associated virus (AAV) Rep protein acts as both a repressor and an activator to regulate AAV transcription during a productive infection. J. Virol. *71*, 1079–1088. https://doi.org/10.1128/jvi.71.2.1079-1088.1997.
21. Schmidt, M., Afione, S., and Kotin, R.M. (2000). Adeno-Associated Virus Type 2 Rep78 Induces Apoptosis through Caspase Activation Independently of p53. J. Virol. *74*, 9441–9450. https://doi.org/10.1128/jvi.74.20.9441-9450.2000.
22. Kondratov, O., Marsic, D., Crosson, S.M., Mendez-Gomez, H.R., Moskalenko, O., Mietzsch, M., Heilbronn, R., Allison, J.R., Green, K.B., Agbandje-McKenna, M., and Zolotukhin, S. (2017). Direct Head-to-Head Evaluation of Recombinant Adeno-associated Viral Vectors Manufactured in Human versus Insect Cells. Mol. Ther. *25*, 2661–2675. https://doi.org/10.1016/j.ymthe.2017.08.003.
23. Blessing, D., Vachey, G., Pythoud, C., Rey, M., Padrun, V., Wurm, F.M., Schneider, B.L., and Déglon, N. (2019). Scalable Production of AAV Vectors in Orbitally Shaken HEK293 Cells. Mol. Ther. Methods Clin. Dev. *13*, 14–26. https://doi.org/10.1016/j.omtm.2018.11.004.
24. van Oers, M.M. (2011). Opportunities and challenges for the baculovirus expression system. J. Invertebr. Pathol. *107*, S3–S15. https://doi.org/10.1016/j.jip.2011.05.001.
25. Wu, C.P., Chang, C.-J., Li, C.-H., and Wu, Y.-L. (2021). The influence of serial passage on the stability of an exogenous gene expression in recombinant baculovirus. Entomol. Res. *51*, 168–175. https://doi.org/10.1111/1748-5967.12500.
26. Rohrmann, G.F. (2019). Baculovirus Molecular Biology, 4th ed. (National Center for Biotechnology Information (US)).
27. Power, J.F., Reid, S., Radford, K.M., Greenfield, P.F., and Nielsen, L.K. (1994). Modeling and optimization of the baculovirus expression vector system in batch suspension culture. Biotechnol. Bioeng. *44*, 710–719. https://doi.org/10.1002/bit.260440607.
28. Smith, R.H., Levy, J.R., and Kotin, R.M. (2009). A simplified baculovirus-AAV expression vector system coupled with one-step affinity purification yields high-titer rAAV stocks from insect cells. Mol. Ther. *17*, 1888–1896. https://doi.org/10.1038/mt.2009.128.
29. Destro, F., Joseph, J., Srinivasan, P., Kanter, J.M., Neufeld, C., Wolfrum, J.M., Barone, P.W., Springs, S.L., Sinskey, A.J., Cecchini, S., et al. (2023). Mechanistic modeling explains the production dynamics of recombinant adeno-associated virus with the baculovirus expression vector system. Mol. Ther. Methods Clin. Dev. *30*, 122–146. https://doi.org/10.1016/j.omtm.2023.05.019.
30. van Lier, F.L., van den End, E.J., de Gooijer, C.D., Vlak, J.M., and Tramper, J. (1990). Continuous production of baculovirus in a cascade of insect-cell reactors. Appl. Microbiol. Biotechnol. *33*, 43–47. https://doi.org/10.1007/BF00170567.
31. Schaly, S., Ghebretatios, M., and Prakash, S. (2021). Baculoviruses in Gene Therapy and Personalized Medicine. Biologics. *15*, 115–132. https://doi.org/10.2147/BTT.S292692.
32. Hong, M., Li, T., Xue, W., Zhang, S., Cui, L., Wang, H., Zhang, Y., Zhou, L., Gu, Y., Xia, N., and Li, S. (2022). Genetic engineering of baculovirus-insect cell system to improve protein production. Front. Bioeng. Biotechnol. *10*, 994743. https://doi.org/10.3389/fbioe.2022.994743.

33. Muzyczka, N. (1992). Use of adeno-associated virus as a general transduction vector for mammalian cells. Curr. Top. Microbiol. Immunol. *158*, 97–129. https://doi.org/10.1007/978-3-642-75608-5_5.

34. Chen, Y., Hu, S., Lee, W., Walsh, N., Iozza, K., Huang, N., Preston, G., Drouin, L.M., Jia, N., Deng, J., et al. (2024). A Comprehensive Study of the Effects by Sequence Truncation within Inverted Terminal Repeats (ITRs) on the Productivity, Genome Packaging, and Potency of AAV Vectors. Microorganisms *12*, 310. https://doi.org/10.3390/microorganisms12020310.

35. Savy, A., Dickx, Y., Nauwynck, L., Bonnin, D., Merten, O.-W., and Galibert, L. (2017). Impact of Inverted Terminal Repeat Integrity on rAAV8 Production Using the Baculovirus/Sf9 Cells System. Hum. Gene Ther. Methods *28*, 277–289. https://doi.org/10.1089/hgtb.2016.133.

36. Li, X., Miller, L.M., Chrzanowski, M., Tian, J., Jarrold, M.F., Herzog, R.W., Xiao, W., Draper, B., and Zhang, J. (2023). Quantitative analysis of preferential utilization of AAV ITR as the packaging terminal signal. Front. Bioeng. Biotechnol. *11*, 1327433. https://doi.org/10.3389/fbioe.2023.1327433.

37. Destro, F., and Braatz, R.D. (2024). Efficient Simulation of Viral Transduction and Propagation for Biomanufacturing. ACS Synth. Biol. *13*, 3173–3187. https://doi.org/10.1021/acssynbio.4c00227.

OMTA, Volume *34*

Supplemental information

Continuous production of recombinant adeno-associated virus in the insect cell/baculovirus expression vector system

John Joseph, Francesco Destro, Arella Yuan, Daniel Antov, Wenyu Chen, Sally Song, Matthew Theriault, Chengcheng Yuan, Alexander Sansom, Chiara Lundin, Tyler Burns, Hadeel Abubaker Hamed, Piero Morales, Jessica Xinyuan Fan, Avner Romero Rodriguez, Sanyambe Sichuma, Courtney McCracken, Caleb Neufeld, Jacqueline M. Wolfrum, Prasanna Srinivasan, Paul W. Barone, Anthony J. Sinskey, Richard D. Braatz, and Stacy L. Springs

Additional information on the mechanistic model of baculovirus infection and propagation

The system states are summarized in Table S1. The description of all model parameters is reported in Table S2. The model equations for the concentrations of cellular species are

$$\frac{dT}{dt} = \mu T - k_{b,T}T(V_1 + V_2) - k_{d,T}T + D(T_{\text{in}} - T) \quad \text{(S1)}$$

$$\frac{\partial i_1}{\partial t} + \frac{\partial i_1}{\partial \tau_1} = k_{b,T}TV_1\delta(\tau_1) - i_1\left(\bar{k}_{d,I_1} + D + \bar{k}_{b,I_1}V_2\right) \quad \text{(S2)}$$

$$\frac{\partial i_2}{\partial t} + \frac{\partial i_2}{\partial \tau_2} = \mathrm{k}_{b,T}TV_2\delta(\tau_2) - i_2\left(\bar{k}_{d,I_2} + D + \bar{k}_{b,I_2}V_1\right) \quad \text{(S3)}$$

$$\frac{\partial c}{\partial t} + \frac{\partial c}{\partial \tau_1} + \frac{\partial c}{\partial \tau_2} = \bar{k}_{b,I_2}i_2V_1 + \bar{k}_{b,I_1}i_1V_2 - c\left(\bar{k}_{d,C} + D\right) \quad \text{(S4)}$$

$$\frac{dW}{dt} = k_{d,T}T + \int_0^\infty \bar{k}_{d,I_1}\, i_1\, d\tau_1 + \int_0^\infty \bar{k}_{d,I_2}\, i_2\, d\tau_2 + \int_0^\infty \int_0^\infty \bar{k}_{d,c}\, c\, d\tau_1 d\tau_2 - WD, \quad \text{(S5)}$$

where D is the dilution rate (equal to the reciprocal of the residence time), τ_1 is the infection age with respect to virus 1, τ_2 is the infection age with respect to virus 2, $\delta(\cdot)$ is the Dirac delta function, the equivalent binding kinetic parameters are calculated based on the cell infection age as

$$\bar{k}_{b,I_j}(\tau_j) = \begin{cases} k_{b,T}, & \text{for } \tau_j < \tau_b \\ k_{b,T}\exp\left(-\beta_b(\tau_j - \tau_b)\right), & \text{for } \tau_j \geq \tau_b \end{cases}, \text{for } j = \{1,2\} \quad \text{(S6)}$$

$$\bar{k}_{b,C}(\tau_1, \tau_2) = \begin{cases} k_{b,T}, & \text{for } \max\{\tau_1, \tau_2\} < \tau_b \\ k_{b,T}\exp\left(-\beta_b(\max\{\tau_1, \tau_2\} - \tau_b)\right), & \text{for } \max\{\tau_1, \tau_2\} \geq \tau_b \end{cases} \quad \text{(S7)}$$

and the equivalent death kinetic parameters are calculated as

$$\bar{k}_{d,I_j}(\tau_j) = \begin{cases} k_{d,T}, & \text{for } \tau_j < \tau_b \\ k_{d,T}\ln\left(\frac{n_{I_j}}{i_j}\right), & \text{for } \tau_j \geq \tau_b \end{cases}, \text{for } j = \{1,2\} \quad \text{(S8)}$$

$$\bar{k}_{d,C}(t, \tau_1, \tau_2) = \begin{cases} k_{d,T}, & \text{for } \max\{\tau_1, \tau_2\} < \tau_d \\ k_{d,T}\ln\left(\frac{n_{C,V_1}(t,\tau_1,\tau_2) + n_{C,V_2}(t,\tau_1,\tau_2)}{c(t,\tau_1,\tau_2)}\right), & \text{for } \max\{\tau_1, \tau_2\} \geq \tau_d \end{cases}. \quad \text{(S9)}$$

The model equations for the free virion concentrations assume that passage N baculovirus (virus 1) is present in the feed with concentration $V_{1,\mathrm{in}}$ and that all infected cells produce only baculovirus of passage greater than N (virus 2):

$$\begin{aligned}\frac{dV_1}{dt} = & -\int_0^{\infty} V_1 \bar{k}_{b,I_1}\, i_1\, d\tau_1 - \int_0^{\infty} V_1 \bar{k}_{b,I_2} i_2\, d\tau_2 - \int_0^{\infty}\int_0^{\infty} V_1 \bar{k}_{b,C} c\, d\tau_1 d\tau_2 \\ & - V_1\left(k_{b,T}T + k_{d,V} + D - V_{1,\mathrm{in}} D\right)\end{aligned} \quad \text{(S10)}$$

$$\begin{aligned}\frac{dV_2}{dt} = & \int_0^{\infty} \left(\bar{k}_{v,I_1} - V_2 \bar{k}_{b,I_1}\right) i_1\, d\tau_1 + \int_0^{\infty} \left(\bar{k}_{v,I_2} - V_2 \bar{k}_{b,I_2}\right) i_2\, d\tau_2 \\ & + \int_0^{\infty}\int_0^{\infty} \left(\bar{k}_{v,C} - V_2 \bar{k}_{b,C}\right) c\, d\tau_1 d\tau_2 - V_2\left(k_{b,T}T + k_{d,V} + D\right).\end{aligned} \quad \text{(S11)}$$

The equivalent kinetic parameters for progeny release account for the infection-age dependency of baculovirus budding are

$$\bar{k}_{v,I_j}(\tau_j) = \begin{cases} 0, & \text{for } \tau_j < \tau_v^{\mathrm{on}} \vee \tau_j > \tau_v^{\mathrm{off}} \\ k_v, & \text{for } \tau_v^{\mathrm{on}} \le \tau_j \le \tau_v^{\mathrm{off}} \end{cases}, \text{for } j = \{1,2\} \quad \text{(S12)}$$

$$\bar{k}_{v,C}(\tau_1,\tau_2) = \begin{cases} 0, & \text{for } \max\{\tau_1,\tau_2\} < \tau_v^{\mathrm{on}} \vee \max\{\tau_1,\tau_2\} > \tau_v^{\mathrm{off}} \\ & k_v, \text{for } \tau_v^{\mathrm{on}} \le \max\{\tau_1,\tau_2\} \le \tau_v^{\mathrm{off}} \end{cases}. \quad \text{(S13)}$$

The model equations for the concentrations of virus bound to cells and of viral genomes in the nucleus of infected cells are

$$\frac{\partial b_{I_1}}{\partial t} + \frac{\partial b_{I_1}}{\partial \tau_1} = V_1\left(k_{b,T}T\,\delta(\tau_1) + \bar{k}_{b,I_1} i_1\right) - b_{I_1}\left(\bar{k}_{d,I_1} + rD + \bar{k}_{b,I_1} V_2 + k_i\right) \quad \text{(S14)}$$

$$\frac{\partial b_{I_2}}{\partial t} + \frac{\partial b_{I_2}}{\partial \tau_2} = V_2\left(k_{b,T}T\,\delta(\tau_2) + \bar{k}_{b,I_2} i_2\right) - b_{I_2}\left(\bar{k}_{d,I_2} + rD + \bar{k}_{b,I_2} V_1 + k_i\right) \quad \text{(S15)}$$

$$\begin{aligned}\frac{\partial b_{C,V_1}}{\partial t} + \frac{\partial b_{C,V_1}}{\partial \tau_1} + \frac{\partial b_{C,V_1}}{\partial \tau_2} & \\ = \bar{k}_{b,I_1} V_2 b_{I_1} \delta(\tau_2) + \bar{k}_{b,I_2} V_1 i_2\, \delta(\tau_1) + \bar{k}_{b,C} c V_1 & \\ - b_{C,V_1}\left(\bar{k}_{d,C} + rD + \bar{k}_{i,C}\right) &\end{aligned} \quad \text{(S16)}$$

$$\begin{aligned}\frac{\partial b_{C,V_2}}{\partial t} + \frac{\partial b_{C,V_2}}{\partial \tau_1} + \frac{\partial b_{C,V_2}}{\partial \tau_2} & \\ = \bar{k}_{b,I_2} V_2 b_{I_2}\, \delta(\tau_1) + \bar{k}_{b,I_1} V_2 i_1\, \delta(\tau_2) + \bar{k}_{b,C} c V_2 & \\ - b_{C,V_2}\left(\bar{k}_{d,C} + rD + \bar{k}_{i,C}\right) &\end{aligned} \quad \text{(S17)}$$

$$\frac{\partial n_{I_1}}{\partial t} + \frac{\partial n_{I_1}}{\partial \tau_1} = \eta k_i b_{I_1} + \bar{k}_{r,I_1} n_{I_1} - n_{I_1}\left(\bar{k}_{d,I_1} + k_{d,N} + rD + \bar{k}_{b,I_1} V_2\right) \tag{S18}$$

$$\frac{\partial n_{I_2}}{\partial t} + \frac{\partial n_{I_2}}{\partial \tau_2} = \eta k_i b_{I_2} + \bar{k}_{r,I_2} n_{I_2} - n_{I_2}\left(\bar{k}_{d,I_2} + k_{d,N} + rD + \bar{k}_{b,I_2} V_1\right) \tag{S19}$$

$$\frac{\partial n_{C,V_1}}{\partial t} + \frac{\partial n_{C,V_1}}{\partial \tau_1} + \frac{\partial n_{C,V_1}}{\partial \tau_2} = \eta k_i b_{C,V_1} + \bar{k}_{r,C}\, n_{C,V_1} + \bar{k}_{b,I_1} n_{I_1} V_2 \delta(\tau_2) - n_{C,V_1}\left(\bar{k}_{d,C} + k_{d,N} + rD\right) \tag{S20}$$

$$\frac{\partial n_{C,V_2}}{\partial t} + \frac{\partial n_{C,V_2}}{\partial \tau_1} + \frac{\partial n_{C,V_2}}{\partial \tau_2} = \eta k_i b_{C,V_2} + \bar{k}_{r,C}\, n_{C,V_2} + \bar{k}_{b,I_2} n_{I_2} V_1 \delta(\tau_1) - n_{C,V_2}\left(\bar{k}_{d,C} + k_{d,N} + rD\right), \tag{S21}$$

where the equivalent kinetic parameters for viral replication are

$$\bar{k}_{r,I_j}(\tau_j) = \begin{cases} 0, & \text{for } \tau_j < \tau_r^{\text{on}} \vee \tau_j > \tau_r^{\text{off}} \\ k_r, & \text{for } \tau_r^{\text{on}} \le \tau_j \le \tau_r^{\text{off}} \end{cases}, \text{for } j = \{1,2\} \tag{S22}$$

$$\bar{k}_{r,C}(\tau_1, \tau_2) = \begin{cases} 0, & \text{for } \max\{\tau_1, \tau_2\} < \tau_r^{\text{on}} \vee \max\{\tau_1, \tau_2\} > \tau_r^{\text{off}} \\ k_r, & \text{for } \tau_r^{\text{on}} \le \max\{\tau_1, \tau_2\} \le \tau_r^{\text{off}} \end{cases}. \tag{S23}$$

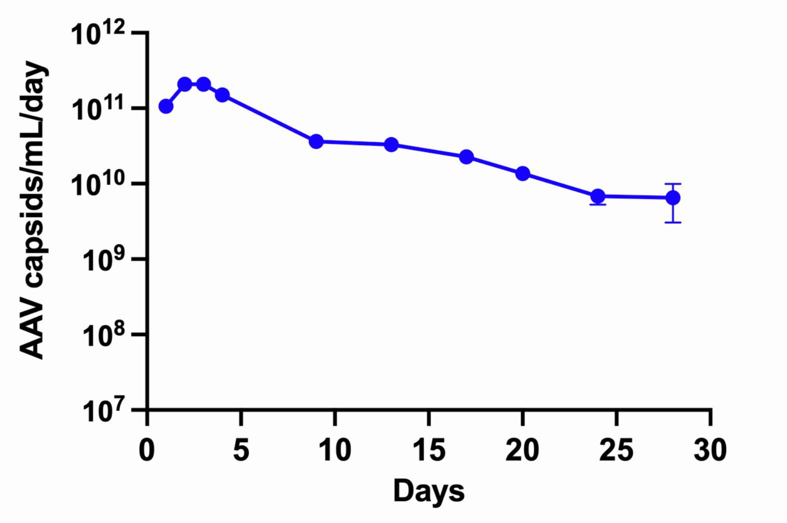

Figure S1. Total capsid titer in the experiment of continuous rAAV production in the IC/BEVS with a two-tank cascade (residence time of 60 hours per tank and no continuous rBV feed).

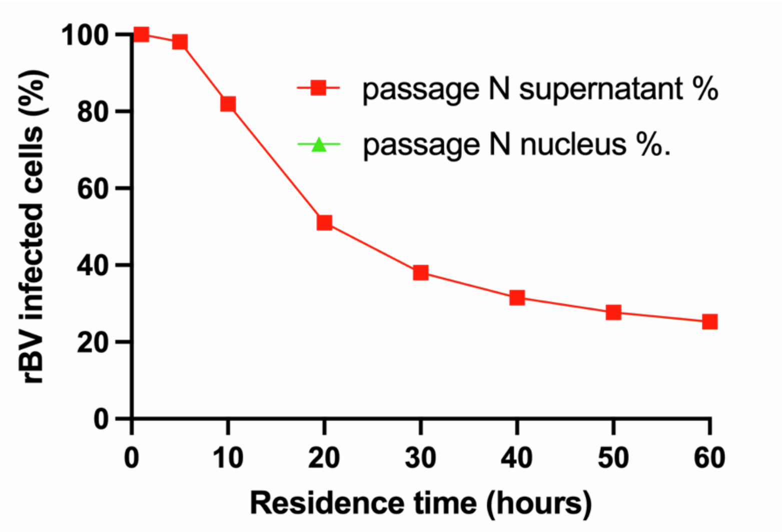

Figure S2. Mechanistic model prediction of the baculovirus passage distribution in the infection reactor at steady-state as a function of the residence time. The reported results represent the fraction of passage *N* (i.e., the passage supplied in the feed) among (i) total virions in the supernatant and (ii) viral genomes in the nuclei of infected cells. The two predictions are numerically very similar, and their curves overlap. A fixed feed of 2.5 million uninfected cells/mL and 2 PFU/cell is considered in the model simulation.

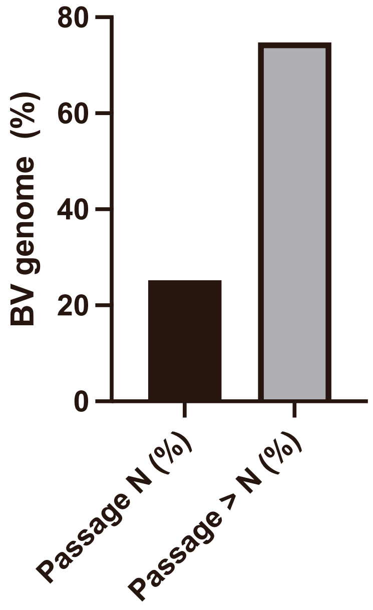

Figure S3. Mechanistic model prediction of the fraction of passage N virus (i.e., the passage supplied in the feed) in the bioreactor at steady state. The distribution is nearly identical for total virions in the supernatant and viral genomes in the nuclei of infected cells. Simulation conditions: 60-hour residence time and feed of 2.5 million uninfected cells/mL and 2 PFU/cell.

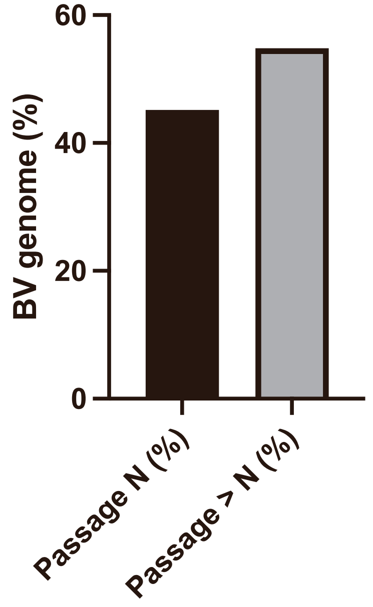

Figure S4. Mechanistic model prediction of the fraction of passage N (i.e., the passage supplied in the feed). The distribution is nearly identical for total

virions in the supernatant and viral genomes in the nuclei of infected cells. Simulation conditions: 16-hour residence time and feed of 2.5 million uninfected cells/mL and 1 PFU/cell.

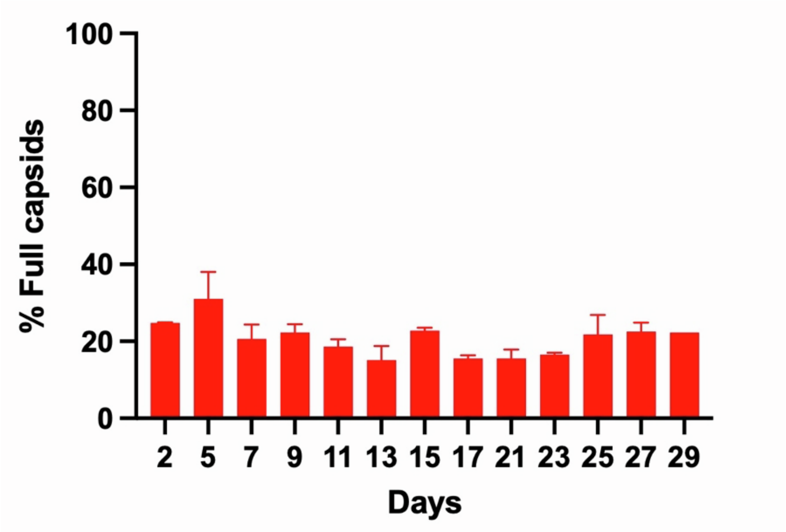

Figure S5. Full capsid percentage quantified during continuous AAV production over 4 weeks using ddPCR and ELISA. Data are presented as mean ± standard deviation.

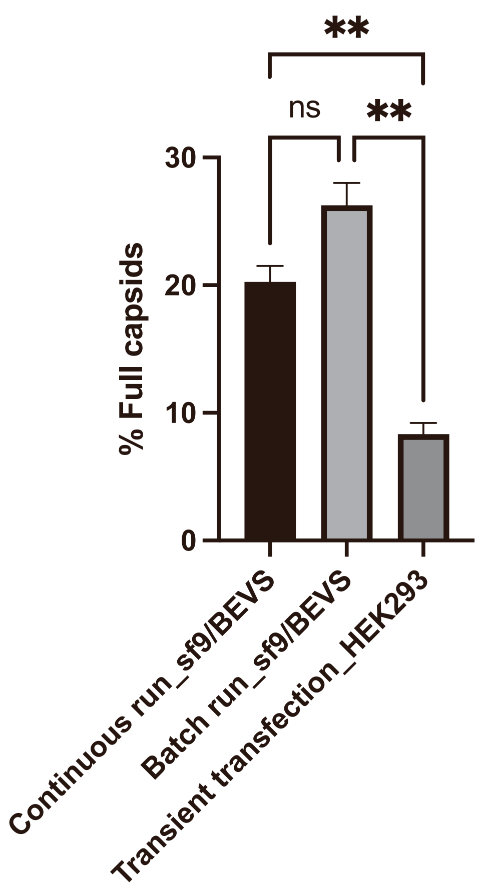

Figure S6. Comparison of full capsid percentage in continuous bioreactor production and batch run experiments. Both Sf9/BEVS-based conditions showed significantly higher percentages of full capsids than transient transfection in HEK293 cells ($p < 0.01$). Data are presented as mean ± standard deviation.

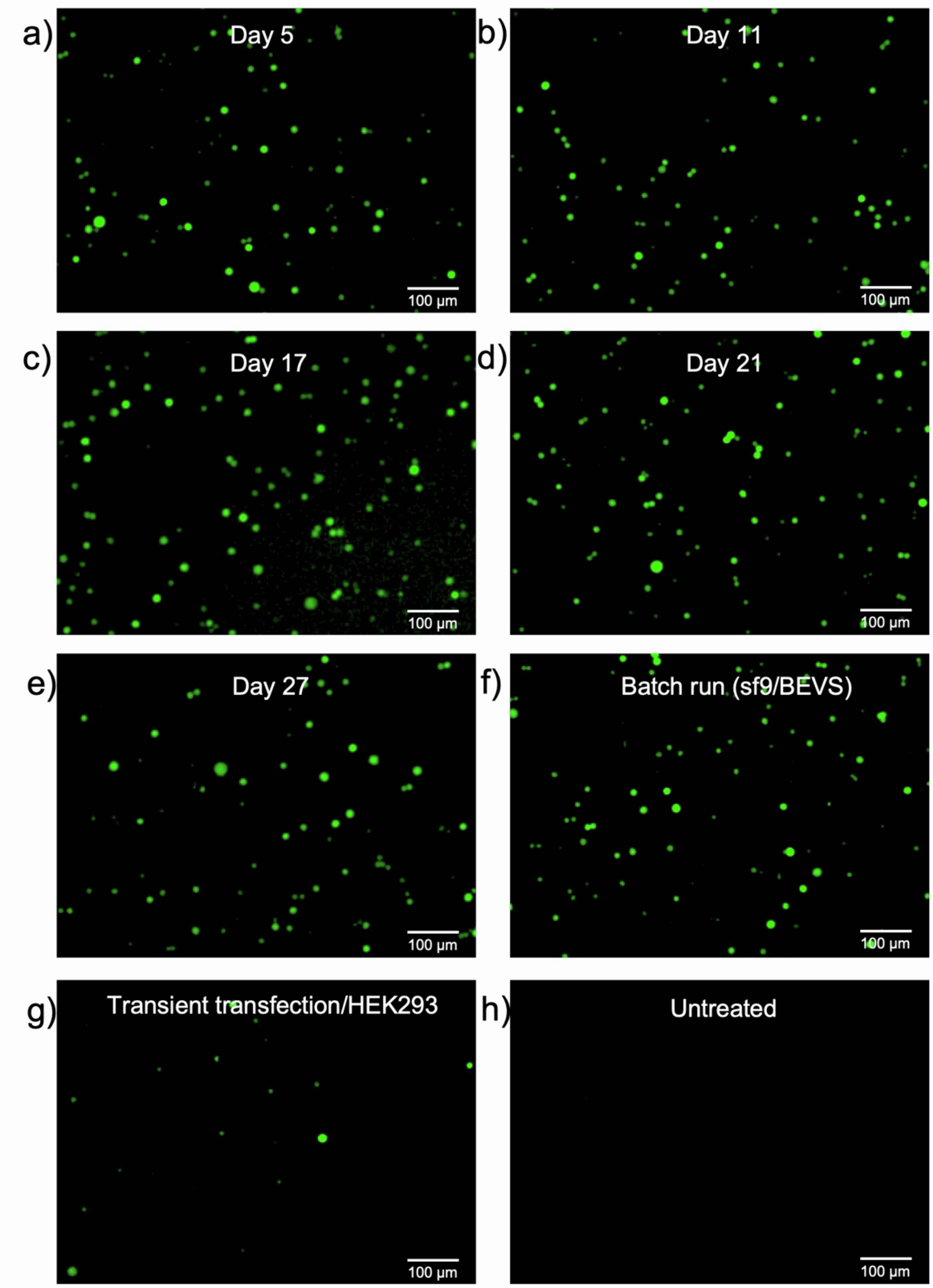

Figure S7. GFP expression in AAV transduced HEK293 cells. Fluorescence images were captured 72 hours post infection for rAAV vectors produced from a-e) continuous production, f) batch run with Sf9/BEVS platform, g) Transient transfection from HEK293 cells and h) untreated cells.

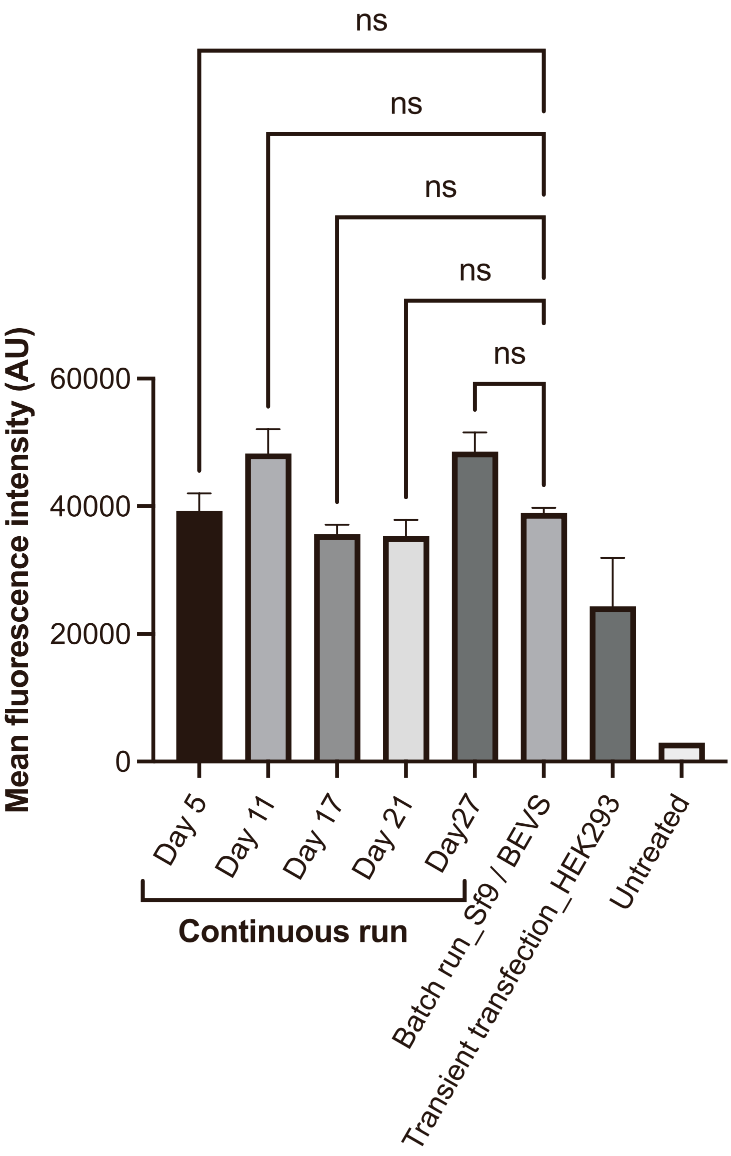

Figure S8. GFP expression quantified in HEK293 cells transduced with rAAV vectors collected during continuous production (Days 7,15, 21 and 27) and batch mode using sf9/BEVS. The control group represents non-transduced HEK293 cells.

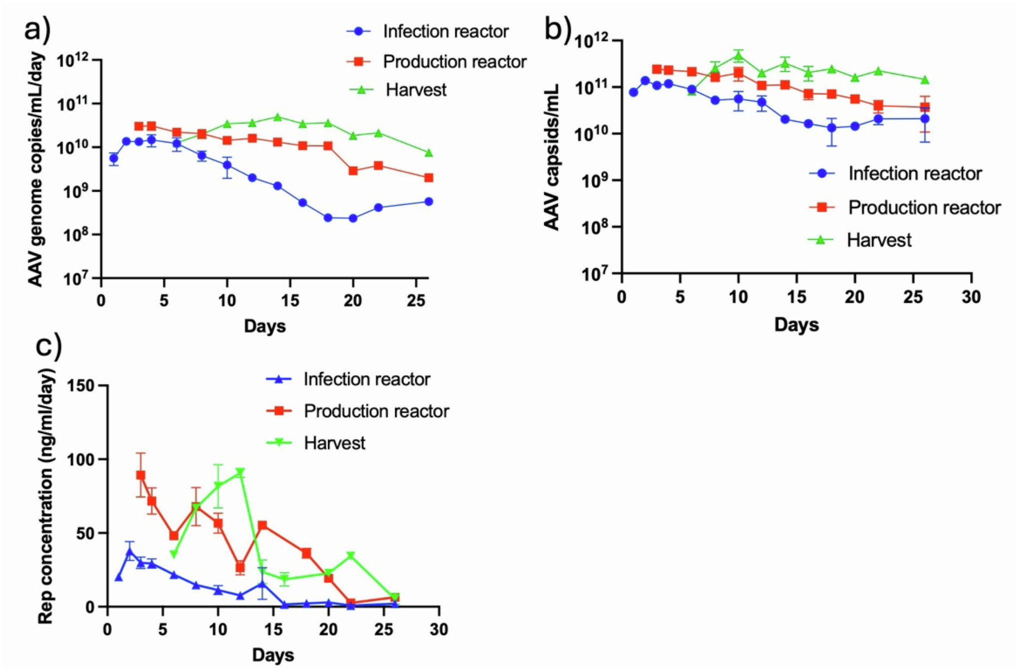

Figure S9. Continuous rAAV production in a three-tank cascade without rBV feed. a) and b) total AAV genome and capsid titers were determined from the cell suspension using ddPCR and ELISA, c) Total Rep protein (Rep72 and Rep52) concentrations were obtained from the cell suspension using ELISA. An average residence time of 60 hours was maintained across all three reactors to provide adequate time to self-replicate insect cells and rBV in the growth and infection reactors, respectively. The maximum rAAV yield was achieved in the production reactor.

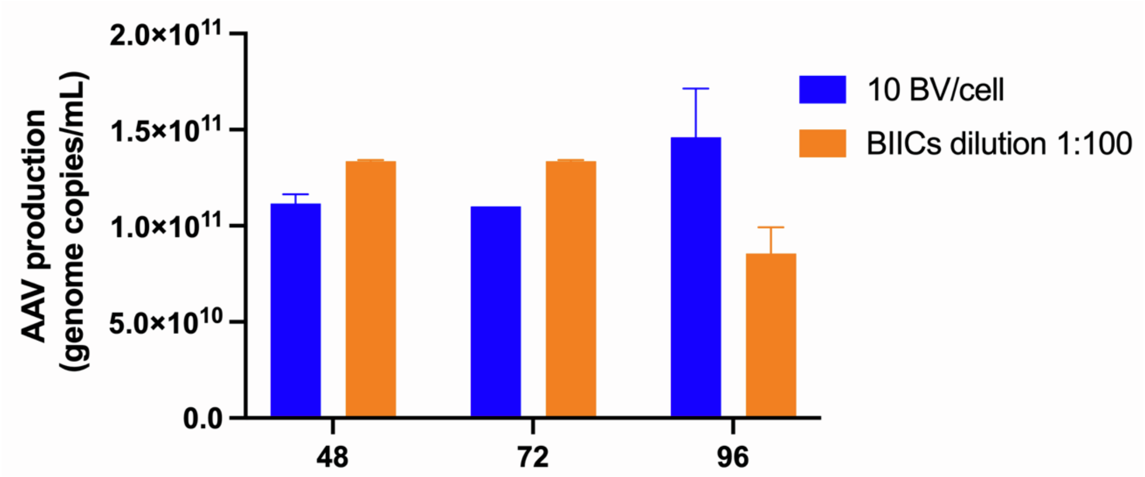

Figure S10. Comparison of budded rBV with BIICs for rAAV production. Cell lysates were collected at 48, 72, and 96 hours post-infection. rAAV genome titers were quantified after DNase treatment using ddPCR.

Table S1. States of the mechanistic model of baculovirus infection and propagation. Virus 1 represents low-passage baculovirus (passage N, i.e., the passage supplied in the feed), whereas virus 2 denotes higher-passage baculovirus (passage > N) arising during propagation.

Symbol	UOM	Description
$b_{I_j}(t,\tau_j)$	virus mL^{-1} hpi $^{-1}$	Virus j attached to $i_j(t,\tau_j)$, for $j=\{1,2\}$
$b_{C,V_j}(t,\tau_1,\tau_2)$	virus mL^{-1} hpi $^{-2}$	Virus j attached to $c(t,\tau_1,\tau_2)$, for $j=\{1,2\}$
$c(t,\tau_1,\tau_2)$	cell mL^{-1} hpi $^{-2}$	Coinfected cells
$i_j(t,\tau_j)$	cell mL^{-1} hpi $^{-1}$	Cells infected by virus j, for $j=\{1,2\}$
$n_{I_j}(t,\tau_j)$	vg mL^{-1} hpi $^{-1}$	Viral genome j in nucleus of $i_j(t,\tau_j)$, for $j=\{1,2\}$
$n_{C,V_1}(t,\tau_1,\tau_2)$	vg mL^{-1} hpi $^{-2}$	Viral genome j in nucleus of $c(t,\tau_1,\tau_2)$, for $j=\{1,2\}$
$T(t)$	cell mL^{-1}	Uninfected cells
$V_j(t)$	virus mL^{-1}	Extracellular virus j, for $j=\{1,2\}$
$W(t)$	cell mL^{-1}	Nonviable cells

Table S2. Parameters of the mechanistic model of baculovirus infection and propagation.

Symbol	UOM	Value	95% Confidence Interval	Description	Source
Infection					
$k_{b,T}$	mL cell^{-1} h^{-1}	6.3×10^{-7}	$(5.2\times10^{-7}, 7.4\times10^{-7})$	Viral binding kinetic constant	Destro et al. (2023)
τ_b	hpi	1.80	(1.3, 2.3)	Onset of viral binding decay for infected cells	Destro et al (2023); Rohrmann (2019)
β_b	[–]	0.5	–	Coefficient for viral binding decay for infected cells	Destro and Braatz (2024)
Cell growth and death					
μ	h^{-1}	2.8×10^{-2}	$(2.7\times10^{-2}, 2.9\times10^{-2})$	Growth kinetic constant for uninfected cells	Mena et al. (2010); Power et al. (1994)
$k_{d,T}$	h^{-1}	6.3×10^{-7}	$(6\times10^{-5}, 1\times10^{-4})$	Death kinetic constant for uninfected cells	Power et al. (1994); Rohrmann (2019)
$k_{d,I}$	h^{-1}	2.9×10^{-3}	$(2.9\times10^{-3}, 1\times10^{-4})$	Death kinetic constant for infected cells	Destro et al. (2023)
τ_d	hpi	24	(22, 26)	Onset of death rate increase for infected cells	Destro et al. (2023); Rohrmann (2019)
Viral degradation					
$k_{d,V}$	h^{-1}	7×10^{-3}	$(1\times10^{-3}, 1.3\times10^{-2})$	Degradation kinetic constant for virions	Power et al. (2024)
$k_{d,N}$	h^{-1}	0	–	Degradation kinetic constant for nuclear viral genome	Destro et al (2023)
Viral trafficking and replication					
k_i	h^{-1}	0.6	(0.48, 0.72)	Virus internalization kinetic constant	Dee and Shuler (1997)
η	[–]	0.50	(0.45, 0.55)	Fraction of internalized virus that reaches the nucleus	Dee and Shuler (1997)
k_r	h^{-1}	0.732	(0.651, 0.813)	Viral genome replication kinetic constant	Destro et al (2023)
τ_r^{on}	hpi	6	(5, 7)	Onset of viral genome replication in infected cells	Destro et al (2023); Rohrmann (2019)
τ_r^{off}	hpi	18	(17, 19)	End of viral genome replication in infected cells	Destro et al (2023); Rohrmann (2019)
Progeny release					

k_v	PFU cell h^{-1}	0.65	(0.5, 1.5)	Progeny release rate	Estimated from serial passage experiments data (Figure 3f)
τ_v^{on}	hpi	18	(16, 20)	Onset of progeny release for infected cells	Destro et al (2023); Rohrmann (2019)
τ_v^{off}	hpi	Cell death	–	End of progeny release for infected cells	Destro et al (2023); Rohrmann (2019)
